# Determination of Selected Isoquinoline Alkaloids from *Chelidonium majus*, *Mahonia aquifolium* and *Sanguinaria canadensis* Extracts by Liquid Chromatography and Their In Vitro and In Vivo Cytotoxic Activity against Human Cancer Cells

**DOI:** 10.3390/ijms24076360

**Published:** 2023-03-28

**Authors:** Tomasz Tuzimski, Anna Petruczynik, Tomasz Plech, Barbara Kaproń, Anna Makuch-Kocka, Małgorzata Szultka-Młyńska, Justyna Misiurek, Bogusław Buszewski, Monika Waksmundzka-Hajnos

**Affiliations:** 1 Department of Physical Chemistry, Medical University of Lublin, Chodźki 4a, 20-093 Lublin, Poland; 2 Department of Inorganic Chemistry, Medical University of Lublin, Chodźki 4a, 20-093 Lublin, Poland; 3 Department of Pharmacology, Medical University of Lublin, Chodźki 4a, 20-093 Lublin, Poland; 4 Department of Clinical Genetics, Medical University of Lublin, Radziwiłłowska 11, 20-080 Lublin, Poland; 5 Department of Environmental Chemistry and Bioanalytics, Faculty of Chemistry, Nicolaus Copernicus University, Gagarina 7, 87-100 Torun, Poland

**Keywords:** cytotoxic activity, danio rerio larvae xenograft model, HPLC-DAD, LC-MS/MS, isoquinoline alkaloids, *Chelidonium majus*, *Mahonia aquifolium*, *Sanguinaria canadensis*

## Abstract

The search for new substances with cytotoxic activity against various cancer cells, especially cells that are very resistant to currently used chemotherapeutic agents, such as melanoma cells, is a very important scientific aspect. We investigated the cytotoxic effect of *Chelidonium majus*, *Mahonia aquifolium* and *Sanguinaria canadensis* extracts obtained from different parts of these plants collected at various vegetation stages on FaDu, SCC-25, MCF-7, and MDA-MB-231 cancer cells. Almost all the tested extracts showed higher cytotoxicity against these cancer cells than the anticancer drug etoposide. The highest cytotoxicity against the FaDu, SCC-25, MCF-7 and MDA-MB-231 cancer cell lines was obtained for the *Sanguinaria candensis* extract collected before flowering. The cytotoxicity of extracts obtained from different parts of *Chelidonium majus* collected at various vegetation stages was also evaluated on melanoma cells (A375, G361 and SK-MEL-3). The highest cytotoxic activity against melanoma A375 cells was observed for the *Chelidonium majus* root extract, with an IC50 of 12.65 μg/mL. The same extract was the most cytotoxic against SK-MEL-3 cells (IC50 = 1.93 μg/mL), while the highest cytotoxic activity against G361 cells was observed after exposure to the extract obtained from the herb of the plant. The cytotoxic activity of *Chelidonium majus* extracts against melanoma cells was compared with the cytotoxicity of the following anticancer drugs: etoposide, cisplatin and hydroxyurea. In most cases, the IC50 values obtained for the anticancer drugs were higher than those obtained for the *Chelidonium majus* extracts. The most cytotoxic extract obtained from the root of *Chelidonium majus* was selected for in vivo cytotoxic activity investigations using a Danio rerio larvae xenograft model. The model was applied for the first time in the in vivo investigations of the extract’s anticancer potential. The application of *Danio rerio* larvae xenografts in cancer research is advantageous because of the transparency and ease of compound administration, the small size and the short duration and low cost of the experiments. The results obtained in the xenograft model confirmed the great effect of the investigated extract on the number of cancer cells in a living organism. Our investigations show that the investigated plant extracts exhibit very high cytotoxic activity and can be recommended for further experiments in order to additionally confirm their potential use in the treatment of various human cancers.

## 1. Introduction

Over the last decades, the incidence rates of cancer, especially cutaneous melanoma, have grown significantly [1]. Therefore, the search for new active anticancer compounds is a crucial element of natural product research. Medicinal plants can be candidates for a common alternative for various cancer treatments, especially because approximately 60% of the drugs currently used in the treatment of cancer patients have been isolated from natural products [1].

Isoquinoline alkaloids, a group of plant-derived biologically active compounds, have traditionally been used as anti-inflammatory, antimicrobial and analgesic remedies [2]. Isoquinoline alkaloids and plants containing isoquinoline alkaloids have also been investigated for many years as alternative regimens to complement chemotherapy. In vitro and in vivo experiments have shown that isoquinoline alkaloids exert anticancer effects through cell cycle arrest, apoptosis and autophagy, which lead to cell death. Large amounts of isoquinoline alkaloids are present in *Chelidonium majus*, *Mahonia* sp. and *Sanguinaria canadensis* [2].

For the determination of *Chelidonium majus* extract components, many techniques, such as nuclear magnetic resonance [3], capillary electrophoresis [4], liquid chromatography–mass spectrometry (LC-MS) [3,5,6] and liquid chromatography-diode array detection [7,8] have been applied. For the determination of alkaloids’ content in the extracts obtained from various *Mahonia* species, HPLC-DAD [9,10], LC-MS and LC-MS/MS [11] have been commonly applied. 

The cytotoxicity of *Chelidonium majus* extracts has been frequently investigated in in vitro studies against various cancer cells. For example, the cytotoxic activity of *Chelidonium majus* extract was tested against human epidermoid carcinoma A431 cells [12]. The obtained results indicated that the extract inhibited the proliferation and induced the apoptosis of cancer cells. In another study, a protoberberine fraction of a *Chelidonium majus* extract was found to reduce the viability of human epithelial cervical cancer (HeLa and C33A) cell lines [3]. 

The cytotoxicity of alkaloids isolated from *Chelidonium majus* has been frequently tested against various cancer cell lines. Kulp and Bragina investigated the cytotoxic activity of sanguinarine, allocryptopine, stylopine, chelidonine and protopine using cancer mouse melanoma (B16F10) cells and human breast cancer (MCF7) cell lines [4]. The mixture of the investigated alkaloids at low concentrations and with low exposure times caused the apoptosis of melanoma cells and was weakly toxic to normal cells. Chelidonine and homochelidonine, their dimethoxy analogues isolated from *Chelidonium majus*, induced cell death in a mini-panel of human leukemic (MOLT-4, Jurkat, HL-60 and HEL 92.1.7), lymphoma (Raji and U-937) and quiescent peripheral blood mononuclear (PBMCs) cancer cell lines [13]. 

A major factor responsible for the failure of chemotherapy is the development of multidrug resistance. Both chelidonine and an extract obtained from *Chelidonium majus* were evaluated for their ability to overcome the multidrug resistance of the Caco-2, HepG-2 and HeLa cancer cell lines [5]. The obtained results indicated that multidrug resistance in cancer cells was modulated by chelidonine and the *Chelidonium majus* extract, the effects being due to a down-regulation of ABC-transporter proteins (P-gp, MRP1 and BCRP), metabolic genes (GST, CYP3A4 and PXR) and an up-regulation of caspase family genes. Chelidonine and the plant extract inhibited Pgp/MDR1 activity in Caco-2 and CEM/ADR5000 cells and reversed their doxorubicin resistance. Additionally, chelidonine and the *Chelidonium majus* extract inhibited the activity of the drug-modifying enzymes CYP3A4 and GST. 

Zhang et al. suggested that chelidonine, a major bioactive isoquinoline alkaloid in *Chelidonium majus*, could exhibit antiproliferative, pro-apoptotic, anti-invasive and anti-inflammatory activity due to the suppression of NF-κB activation and NF-κB-regulated gene products [14].

The anticancer activity of the alkaloids obtained from *Chelidonium majus* was also investigated against melanoma. Most of the reports concerned research on berberine’s anticancer activity. The effects of berberine on the migration of human melanoma (A375 and Hs294) cells were examined by Singh et al. [15]. The obtained results indicated that the alkaloid inhibited the migration of the tested cells. The results obtained by Liu et al. also indicated that berberine suppressed the mobility, migration and invasion of human melanoma (A375.S2) cells under in vitro conditions [16]. The effects of berberine on the proliferation, apoptosis and migration of skin melanoma A375 cells were also reported [17]. Other investigations showed that berberine elicited the alteration of protein expression patterns and signaled the induction of the isozymes of catalase and superoxide dismutase in melanoma cells [18]. Kou et al. investigated the effect of berberine on the metastasis of mouse melanoma B16 cells and suggested that berberine could inhibit the invasion and migration of mouse melanoma cells through the inhibition of the epithelial-to-mesenchymal transition-related protein [19]. 

The cytotoxicity of *Chelidonium majus* extracts have been investigated using in vivo experiments much less often. Capistrano et al. investigated the antitumor activity of *Chelidonium majus* [7], both in vitro and in vivo. In vitro experiments performed on human cancer cell lines, i.e., pancreas cancer (PANC-1), colon cancer (HT-29), breast cancer (MDA-MB-231), primary endometrium cancer (PC-EM005 and PC-EM002) and murine pancreatic adenocarcinoma (PANC02), showed a promising cytotoxic activity of the investigated extract, particularly against pancreatic cancer. An in vivo study performed using a highly metastatic murine pancreatic model showed a reduction in the number of metastases compared to the control group [7]. 

Plants of the genus *Mahonia* are used in folk medicine worldwide for the treatment of tuberculosis, dysentery, pharyngolaryngitis, eczema and different skin disorders. They also exhibit antibacterial, antifungal, anti-inflammatory and cytotoxic properties [20]. 

The cytotoxicity of *Mahonia* species extracts and alkaloids isolated from these extracts were tested in vitro against various cancer cells [21,22,23,24,25]. 

The cytotoxic activity of *Mahonia* species extracts has been very rarely investigated under in vivo conditions. The inhibitory effect of *Mahonia oiwakensis* extracts on tumor cell growth was investigated using a nude mouse xenograft model [26]. In A549 tumor-xenografted nude mice, the *Mahonia oiwakensis* extracts were found to inhibit the proliferation and induce the apoptosis of tumor cells.

The cytotoxicity of *Sanguinaria canadensis* extracts, despite the very high content of isoquinoline alkaloids with confirmed cytotoxic activity (e.g., sanguinarine and chelerythrine), have been rarely investigated. Senchina et al. tested the proliferation of immortalized human myelogenous leukemia (K562) cells after treatment with *Sanguinaria canadensis* extracts [27]. Tuzimski et al. investigated the cytotoxicity of some isoquinoline alkaloids and extracts obtained from *Sanguinaria canadensis* collected before, during and after flowering against human melanoma cell lines (A375, G361 and SK-MEL-3) [28].

The aim of this study was to investigate the cytotoxic activity of selected isoquinoline alkaloid standards, *Chelidonium majus*, *Mahonia aquifolium* extracts obtained from various plant parts and *Sanguinaria canadensis* extracts obtained from the whole plant collected at various vegetation stages against the human breast adenocarcinoma (MDA-MB-231), tongue squamous-cell carcinoma (SCC-25), human breast cancer (MCF-7) and hypopharyngeal squamous-cell carcinoma (FaDu) cell lines. An additional objective was to investigate the effect of the isoquinoline alkaloids and extracts obtained from various parts of *Chelidonium majus* on the viability of three melanoma cell lines, i.e., human malignant melanoma cells (A375), the human Caucasian malignant melanoma cell line (G361) and the human malignant melanoma cell line (SK-MEL-3). To the best of our knowledge, the cytotoxicity of *Sanguinaria candensis* extracts on the MCF-7, SCC-25 and FaDu cell lines and the cytotoxicity of *Chelidonium majus* extracts on the G361 and SK-MEL-3 cell lines, as well as the dependence of plant collection season on the cytotoxic activity of these extracts, were investigated for the first time in our study. Another objective of the research was the in vivo determination of the cytotoxicity of *Chelidonium majus* root extract using a *Danio rerio* larvae xenograft model. 

## 2. Results and Discussion

### 2.1. Determination of the Alkaloid Contents in Plant Extracts by HPLC-DAD

Isoquinoline alkaloids were chromatographed on a Polar RP column with a mobile phase containing acetonitrile, water and the ionic liquid 1-butyl-3-methylimidazolium tetrafluoroborate in the gradient system described in the “Experimental Procedure” section. The chromatographic condition was partially based on the previously published method applied for the determination of isoquinoline alkaloids [29]. The currently used gradient allowed for a reduction in the analysis time (from 60 to 30 min), resulting in more symmetrical peaks on the chromatograms and good separation selectivity.

The same chromatographic conditions as used for the analysis of alkaloid standards were developed for the quantitative determination of selected isoquinoline alkaloids in plant extracts. Three plants characterized by a very high content of isoquinoline alkaloids, i.e., *Chelidonium majus*, *Mahonia aquifolium* and *Sanguinaria canadensis*, were selected for the study. The identities of the analyte peaks in the plant extracts were confirmed by the comparison of their retention times and UV spectra with the retention times and spectra of alkaloid standards, as well as by LC-MS analysis. The exemplary chromatograms obtained for *Chelidonium majus* root and herb, *Mahonia aquifolium* cortex and root and *Sanguinaria canadensis* extracts are presented in Figure S1. Large differences were observed in the content of alkaloids in the extracts obtained from various plants and from different parts of these plants, as well as from plant materials collected at various vegetation times (Table 1).

The content of isoquinoline alkaloids in the investigated extracts, especially those obtained from *Chelidonium majus*, has been described in several previous reports. For example, El-Readi et al. identified chelidonine as the main alkaloid in the extract obtained from the aerial parts of *Chelidonium majus* [5]. In our investigations, chelidonine was also present in the highest concentrations in most extracts from *Chelidonium majus*. Kulp et al. determined the isoquinoline content in the *Chelidonium majus* extract obtained from aerial parts collected during flowering by capillary electrophoresis [30]. They determined, inter alia, 0.25 mg/g of dry plant material of chelidonine, 0.55 mg/g of dry plant material of sanquinarine, 0.40 mg/g of dry plant material of chelerythrine and 0.49 mg/g of dry plant material of protopine. These results were the most similar to those obtained in our experiments for extracts from plant material collected during flowering, but the contents of sanguinarine and chelerithrine in our investigations were lower, while those of protopine and chelidonine were higher. Gu et al. determined the following contents in the *Chelidonium majus* extracts obtained from the herb collected in different locations: from 0.57 to 2.42 mg/g of dry plant material of protopine, from 0.73 to 2.34 mg/g of dry plant material of chelidonine, from 0.66 to 2.93 mg/g of dry plant material of sanguinarine and from 0.18 to 2.80 mg/g of dry plant material of chelerythrine [8]. Our results obtained for protopine were in the same range, but for other alkaloids, they were lower for most of the investigated extracts. From 7.8 to 10.7 mM, from 8.8 to 23.3 mM and from 31.3 to 73.2 mM of berberine in crude extract, chloroform and ethanol extracts obtained from *Mahonia aquifolium* stem bark extract were determined, respectively [21]. In the extracts from *Mahonia aquifolium* stem bark, from 11.5 to 15.1, from 12.1 to 27.9 and from 34.2 to 73.8 mM in crude extract, chloroform and ethanol extracts of palmatine were also determined, respectively.

Campbell et al. determined a content from 1.82 to 2.96 mg/g of dry plant material of sanquinarine in the extracts obtained from the rhizomes of *Sanguinaria canadensis* [31]. A higher content of sanguinarine was obtained in our experiments, but the investigated extracts were obtained from whole plants.

### 2.2. LC-MS/MS

The presence of the determined alkaloids in plant extracts was confirmed by LC-MS/MS analysis. Full MS and MS/MS spectra between 40 and 370 *m*/*z* were acquired for each target compound. Moreover, because the ESI mass spectrum was obtained from an acidic solution that put the investigated alkaloids into the positively charged quaternary nitrogen form, their highest mass peaks corresponded to the actual molecular mass, [M+H]^+^.

Table 2 summarizes the chromatographic information and MS-based information (adduct forms, observed mass and product ions). Highly abundant protonated molecule [M+H]^+^ ions of all the studied alkaloids were observed in the ESI mass spectra due to the strong basicity of the secondary or tertiary amine group. Hence, the relevant isoquinoline alkaloids were identified based on MS spectra for berberine (*m*/*z* = 335.7429), chelerythrine (*m*/*z* = 347.7489), chelidonine (*m*/*z* = 354.3920), palmatine (*m*/*z* = 351.7853), magnoflorine (*m*/*z* = 341.7917), protopine (*m*/*z* = 353.7655) and sanguinarine (*m*/*z* = 331.7065). Moreover, their presence in real plant samples regarding different morphological parts (before, after or during flowering) was confirmed by MS/MS spectra and collision-induced dissociation (CID) of the peak of the most intense ion.

In order to optimize the proposed HPLC−MS/MS method, various parameters were examined to achieve the best separation and retention for the analytes. In order to achieve a fast and reliable separation, other parameters such as drying gas temperature (DGT), capillary voltage (CV) and fragmentor voltage (FV) were investigated. The optimal conditions of MS parameters for the determination of target compounds were estimated using Box–Behnken as the experimental design. The 2D contour plots obtained for the investigated alkaloids are presented in Figure S2.

The identification of the investigated alkaloids in plant extracts was based on accurate mass measurements, the isotopic distribution of parent ions and the study of their fragmentation patterns (Figure S3). In the MS/MS spectrum of berberine, the major product ions appear at *m*/*z* = 319.7029 and correspond to the elimination of a methyl radical and CH_4_ from the methoxy substituent. The ion at *m*/*z* = 305.6823 is formed by the continuous elimination of two methyl radicals, and the ion at *m*/*z* = 304.1893 is formed by the loss of CH_3_OH from the precursor ion. The ions at *m*/*z* = 291.6987 and 277.6827 are then formed by the loss of CO from the ions at *m*/*z* = 319.7029 and *m*/*z*=305.6823, respectively. Moreover, this sequential loss of a methyl radical and CO is the characteristic fragmentation pathway of this kind of alkaloid (Shim et al., 2013). This fragmentation behavior also occurs in other analyzed alkaloids, such as palmatine. Additionally, the MS/MS spectra of [M+H]^+^ ions for palmatine are characterized by the presence of the most abundant ion at *m*/*z* = 335.7442 and several fragment ions. These characteristic ions at *m*/*z* = 307.7351, 277.6835 and 244.6977 are formed mainly by the cleavage of substituted methoxyl and methylenedioxyl groups on the A- and D-rings, respectively. In the case of magnoflorine, the signal at *m*/*z* = 296.7147 confirms the loss of two methyl groups and the NH group at the quaternary ammonium ion. Moreover, the signal at *m*/*z* = 264.6899 confirms the detachment of an additional –CH_3_OH group out of the signal at *m*/*z* = 296.7147. The ion at *m*/*z* = 236.7089 shows a subsequent loss of the –CO group out of the signal at 264.6899. In the case of protopine, the fragment ion at *m*/*z* = 205.7380 and 148.8711 in the MS/MS spectra are generated by RDA (retro-Diels-Alder reaction) C-ring opening (Shim et al., 2013), but given the presence of hydroxyl groups, the product ions at m/z = 336.1209 and *m*/*z* = 188.7681 are probably formed by the loss of H_2_O from the molecular ion and from the *m*/*z* = 205.7380 ion. In the case of sanguinarine, the MS/MS spectra exhibited fragment ions at *m*/*z* = 316.6761 [M—CH_3_]^+^, *m*/*z* = 303.6993 [M—CO]^+^ and *m*/*z* = 273.6853 [M—(CH_2_O + CO)]^+^. Based on the obtained results, similar fragmentation could be assumed for chelerythrine at *m*/*z* = 347.7489 and chelidonine at *m*/*z* = 354.3920, which were mainly fragmented to *m*/*z* = 303.6990 and *m*/*z* = 274.6920 (Shim et al., 2013).

### 2.3. Investigation of the In Vitro Cytotoxic Activity of Plant Extracts against MCF-7, SCC-25, MDA-MB-231 and FaDu Cells

The cytotoxic activity of plant extracts was carried out using the following human cancer cell lines: human pharyngeal squamous carcinoma cells (FaDu), human tongue squamous carcinoma cells (SCC-25), human breast adenocarcinoma (MCF-7) and human triple-negative breast adenocarcinoma (MDA-MB-231). The cytotoxicity of alkaloid standards against the same cancer cell lines was investigated in the same conditions and was previously described [29]. The obtained results were expressed as IC_50_ values, which represent the concentrations that are required for a 50% inhibition of cell viability (Table 3). The differences in the cytotoxic activity against the tested cancer cell lines were found for both the extracts obtained from different plants and the extracts obtained from different plant parts. Differences in cytotoxicity were also observed for the extracts prepared from parts of plants collected at different times of plant vegetation. 

The extracts obtained from the *Chelidonium majus* herb exhibited the highest cytotoxic activity against the SCC-25 cell line (IC_50_ = 1.76 μg/mL), while the IC_50_ obtained for the same extract against MDA-MB-231 cells was equal to 25.31 μg/mL. The highest cytotoxicity was also observed against the SCC-25 cell line after exposure to the extracts obtained from the *Chelidonium majus* root collected after flowering (IC_50_ = 0.95 μg/mL). The *Chelidonium majus* pod extract had the highest cytotoxic activity against FaDu cells (IC_50_ = 3.94 μg/mL). The highest cytotoxic properties among *Chelidonium majus* extracts against all tested cancer cell lines were found for the extract obtained from the plant root. 

In most cases, the extracts obtained from various parts of *Mahonia aquifolium* collected during flowering showed lower cytotoxic activity compared to the extracts obtained from *Chelidonium majus* against all tested cancer cell lines. The lowest cytotoxicity of the *Mahonia aquifolium* extracts, except for the extract obtained from the barked stalk, was observed against SCC-25 cells. The highest cytotoxic activity of all the investigated extracts obtained from *Mahonia aquifolium* was against the FaDu cell line. The IC_50_ values obtained on the cell line ranged from 5.73 μg/mL after treatment with the extract obtained from the barked stalk to 46.77 μg/mL after exposure to the extract obtained from the plant leaves. The lowest cytotoxic properties among the *Mahonia aquifolium* extracts were observed for the extract obtained from the leaves, and the highest for the extract obtained from the barked stalk against SCC-25 and FaDu cells, from the cortex against the MCF-7 cell line and from the root against MDA-MB-231 cells. Our study extends the range of the cytotoxic activity of the *Mahonia aquifolium* extracts to previously untested cancer cell lines and confirms their previously reported activity against various cancer cells. The cytotoxic activity of primary and secondary metabolites in the *Mahonia aquifolium* extracts was previously determined against the human cervical adenocarcinoma (HeLa) cell line and correlated with the chemical composition of these extracts [21]. The highest cytotoxic activity was reported for berberine, palmatine and berbamine. The cytotoxic activity of the *Mahonia aquifolium* extract against three cancer cell lines, i.e., DLD-1 colon carcinoma cells, A2780 ovary adenocarcinoma cells and A375 malignant melanoma cells, was reported in [24]. The combination of doxorubicin and *Mahonia aquifolium* extract inhibited the proliferation, migratory potential and invasiveness of malignant (A549) cells [23]. The IC_50_ values for various combinations of doxorubicin and *Mahonia aquifolium* extract were very low (from 0.0036 μg/mL to 0.4457 μg/mL). 

In vitro investigations on the cytotoxicity of various *Mahonia* species against different cancer cell lines have also been described. A *Mahonia fortunei* extract exhibited cytotoxic activity and apoptosis-induction activity against MCF-7 breast cancer cells, the A431 epidermal cell line and the U87-MG glioma cell line [22]. Palmatine isolated from *Mahonia bealei* under in vitro conditions inhibited the proliferation and inflammation processes in the HT-29 and SW-480 human colorectal cancer cell lines [25]. 

A decrease in the viability of all the tested cancer cells was observed after their exposure to the extracts obtained from *Sanguinaria canadensis.* In almost all cases, the IC_50_ values were below 5 μg/mL, but after exposure of MDA-MB-231 cells to the extract obtained from material collected after flowering, the IC_50_ = 11.37 μg/mL. A very high cytotoxicity (IC_50_ < 1.0 μg/mL) was found after exposure of SCC-25 cells to extracts prepared from *Sanguinaria candensis* collected before flowering (IC_50_ = 0.90 μg/mL) and against FaDu cells after exposure to all the investigated extracts from *Sanguinaria candensis*, with the IC_50_ values ranging from 0.21 μg/mL for the extract from plant material collected before flowering to 0.41 μg/mL for the extract from plant material collected after flowering.

The MCF-7 cell lines exhibited the lowest viability after treatment with an extract from *Sanguinaria candensis* collected during flowering, with an IC_50_ of 1.36 μg/mL. A similar IC_50_ value was obtained after exposure of the cells to an extract from *Sanguinaria candensis* collected before flowering (IC_50_ = 1.48 μg/mL). The same two extracts obtained from *Sanguinaria candensis* collected before and during flowering exhibited the most cytotoxic properties against MDA-MB-231 cells (IC_50_ = 4.15 and 4.26 μg/mL, respectively). The viability of SCC-25 was the lowest after exposure to the *Sanguinaria candensis* extract obtained from plant material collected before flowering, with an IC_50_ of 0.90 μg/mL. A similar level of cytotoxic activity was observed against the cell line after treatment with *Chelidonium majus* root extract (IC_50_ = 0.95 μg/mL). 

The cytotoxicity of plant extracts was also compared with the cytotoxic activity of etoposide, an anticancer drug, against the same cancer cell lines. The IC_50_ values obtained for the drug ranged from 38.73 to 223.94 μg/mL and, in almost all cases, were higher than those obtained for the investigated plant extracts. 

Besides the very high cytotoxicity of the extracts obtained from *Sanguinaria candensis*, high cytotoxic activity was also found for the extracts obtained from *Chelidonium majus.* For this reason, in the next step of the experiments, the cytotoxicity of the extracts obtained from the herb and root of *Chelidonium majus* against the same cancer cell lines was investigated and compared with their cytotoxicity against normal human fibroblasts (Table 4). In most cases, the extracts obtained from the root were characterized by higher cytotoxicity compared to the extracts from the herb collected at the same time. Differences in cytotoxicity between the extracts from the root and the herb were especially great against MCF-7, MDA-MB-231 and SCC-25 cells. The *Chelidonium majus* root and herb extracts were prepared from plant materials collected in April, May and June 2018, which corresponded with the plant’s vegetation stages before, during and after flowering. The cytotoxic activity of the extracts varied depending on the month in which the plant material was collected. For example, the cytotoxicity of the extracts obtained from both the root and the herb against MCF-7 cells increased as regards the extract obtained from the plant material collected in April (IC_50_ = 5.46 and 28.23 μg/mL, respectively) relative to the extract obtained from the plant material collected in June (IC_50_ = 0.17 and 11.97 μg/mL, respectively). The lowest viability of MDA-MB-231 cells was observed after exposure to the root and herb extracts obtained from *Chelidonium majus* collected in May, with IC_50_ values of 0.32 and 9.36 μg/mL, respectively. The cytotoxic activity against SCC-25 cells treated with the extract prepared from the root was the highest when the plant material was collected in May (IC_50_ = 0.62 μg/mL), while the highest cytotoxicity among the herb extracts was shown for the extract obtained from the plant material collected in June (IC_50_ = 26.93 μg/mL). The lowest viability of FaDu cells was found after exposure to both the root and herb extracts obtained from *Chelidonium majus* collected in May, with IC_50_ values of 1.96 and 0.14 μg/mL, respectively. It was the only case in our investigations in which the cytotoxic activity of the herb extract was higher than that of the root extract from *Chelidonium majus* collected at the same time. 

To compare the cytotoxicity of the *Chelidonium majus* extracts against normal human fibroblasts, IC_50_ values were calculated (Table 4). In eight cases, the IC_50_ values obtained for the fibroblasts were higher than those obtained after exposure of cancer cells to the same plant extracts. The extract obtained from the plant root collected in May exhibited higher cytotoxicity against MCF-7, MDA-MB-231 and SCC-25 cells (with IC_50_ values of 0.20, 0.32 and 0.62 μg/mL, respectively) than against fibroblasts, with an IC_50_ of 0.67 μg/mL. A higher level of cytotoxicity against MCF-7 cells compared to fibroblasts was found after exposure to the *Chelidonium majus* root and herb extracts obtained from plant material collected in June. The viability of FaDu cells was lower than the viability of fibroblasts after treatment with all the herb extracts. 

### 2.4. Investigation of In Vitro Anticancer Activity of Chelidonium majus Extracts against A375, SK-MEL-3 and G361 Cells

The cytotoxic activity of the extracts obtained from different parts of *Chelidonium majus* collected after flowering was investigated using human malignant melanoma cells (A375), the human Caucasian malignant melanoma cell line (G361) and the human malignant melanoma cell line (SK-MEL-3). Table 5 summarizes the IC_50_ values obtained from the melanoma cells. The lowest cytotoxic activity against all the tested cell lines was found for the extract from seeds. The IC_50_ values obtained after exposure to the extract ranged from 6.97 μg/mL for the G361 cells to >200 μg/mL for the A375 cells. Accordingly, the lowest concentrations of isoquinoline alkaloids especially exhibited higher cytotoxicity, in particular for sanguinarine and chelerythrine (only about 0.002 mg/g of dry plant material). The total investigated alkaloid content was also the lowest (0.094 mg/g of dry plant material). A higher level of cytotoxicity was observed for the extract obtained from seed pods. The highest IC_50_ value, about 180 μg/mL, was obtained for the A375 cells, and the lowest for the G361 cells (IC_50_ = 5.77 μg/mL). The extract contained only slightly higher contents of most cytotoxic alkaloids, but the total isoquinoline alkaloid content was higher (1.630 mg/g of dry plant material). A higher level of cytotoxic activity was observed after treating all the tested melanoma cells with the extract obtained from the *Chelidonium majus* herb. The lowest viability was found for G361 cells, with an IC_50_ of 2.27 μg/mL, and the highest for A375 cells, with an IC_50_ of 27.91 μg/mL. The extract exhibited the most cytotoxic activity against the G361 cell line. In the extract, 2.118 mg of isoquinoline alkaloids per gram of dry plant material was determined. The lowest viability of the A375 and SK-MEL-3 cells was observed after exposure to the extract obtained from the root, with IC_50_ values of 12.65 and 1.93 μg/mL, respectively. The highest cytotoxicity of the extract was correlated with the highest total alkaloid content (4.585 mg/g of dry plant material). 

### 2.5. Investigation of In Vitro Anticancer Activity of Chelidonium majus Extracts by BrdU Incorporation

The antiproliferative effect of the most active *Chelidonium majus* extracts was also investigated on all the tested cell lines using the BrdU assay, which indicates the rate of incorporation of 5′-bromo-2′-deoxy-uridine (BrdU) during DNA biosynthesis (Table 4 and Table 5). The extracts from *Chelidonium majus* inhibited BrdU incorporation dose-dependently in the tested cells. The obtained results indicated that all the investigated extracts exhibited antiproliferative activities. The *Chelidonium majus* root and herb extracts showed a very high antiproliferative effect, which confirms their anticancer potential. A 50% inhibition of G361 cells was observed at 4.85 µg/mL of cisplatin and from 3.98 µg/mL for the *Chelidonium majus* root extract to 5.17 µg/mL for the *Chelidonium majus* seed extract obtained from plant material collected after flowering. The extracts obtained from the *Chelidonium majus* roots and herb also exhibited higher antiproliferative activity against SK-MEL-3 cells (IC_50_ = 1.17 and 3.62 µg/mL, respectively) compared to the activity of cisplatin (IC_50_ = 7.58 µg/mL). The results of the in vitro cytotoxicity investigations using both assays indicated that the *Chelidonium majus* extracts exhibited a high antiproliferative effect and confirmed their anticancer potential.

### 2.6. Correlation of Some Alkaloid Contents with the Cytotoxic Activity of Chelidonium majus Extracts

The content of alkaloids that showed strong cytotoxic properties (chelerythrine and sanguinarine) in the extracts obtained from various parts of *Chelidonium majus* collected at different vegetation stages was also correlated with the IC_50_ values obtained for these extracts (Table 6). The highest correlation coefficient values obtained on all the tested cell lines were observed for chelerythrine (correlation coefficients (r) ranging from 0.5340 obtained from the correlation with IC_50_ on FaDu cells to 0.8845 obtained from the correlation with IC_50_ on MCF-7). Slightly weaker correlations were obtained for sanguinarine (r from 0.5065 to 0.8608). Good correlations were obtained for the sum of both alkaloid contents (r from 0.5293 to 0.8835). When correlating the IC_50_ values with the content of other determined isoquinoline alkaloids, which exhibited lower cytotoxicity, lower values of r were obtained. The high r values obtained in most cases from the correlations with the contents of the most cytotoxic alkaloids can indicate a very strong influence of the isoquinoline alkaloids chelerythrine and sanguinarine on the cytotoxic properties of the *Chelidonium majus* extracts. The investigated extracts also contain other alkaloids not determined in our investigations, which may also influence their cytotoxic properties. For this reason, further research on both the composition and related cytotoxic properties is advisable.

### 2.7. Comparison of the In Vitro Cytotoxic Activity of Chelidonium majus Extracts with Cytotoxic Activity of Anticancer Drugs

To compare the very high cytotoxic activity of the extracts obtained from different parts of *Chelidonium majus*, IC_50_ values were also determined in the same conditions for three anticancer drugs: etoposide, cisplatin and hydroxyurea (Table 5). The lowest cytotoxic activity against the tested melanoma cell lines was observed after exposure to hydroxyurea (IC_50_ > 200 μg/mL), while the highest activity was observed after exposure to cisplatin, with IC_50_ values ranging from 10.62 μg/mL obtained after treatment of the A375 cells to 14.42 obtained for the SK-MEL-3 cells. In most cases, the cytotoxic activity of the investigated plant extracts against the tested melanoma cell lines was higher than that observed for the anticancer drugs. Higher IC_50_ values were obtained only after exposure to all the investigated extracts compared with the IC_50_ value after exposure of the A375 cells to cisplatin. However, the extracts obtained from the plant’s herb and root were more cytotoxic than the other two drugs. The lowest IC_50_ values were obtained after exposure of the G361 cells to all the investigated extracts (IC_50_ from 2.27 to 6.97 μg/mL) compared to the IC_50_ values obtained for all the tested anticancer drugs (IC_50_ from 11.53 to >200 μg/mL). The extracts obtained from the herb and root of *Chelidonium majus* were also more cytotoxic (IC_50_ = 7.89 and 1.93 μg/mL, respectively) than all the tested drugs (IC_50_ from 14.42 to >200 μg/mL) against the SK-MEL-3 cells. 

### 2.8. In Vivo Investigations of Toxicity of Chelidonium majus Root Extract

*The Chelidonium majus* root extract collected after flowering exhibited the highest cytotoxicity against most of the investigated cancer cell lines in in vitro experiments and was selected for the further in vivo study. For these experiments, a *Danio rerio* larval xenograft model was selected due to the short duration of the experiments, the small larval size, the ease of administration of the tested compound, the transparency of the larvae and, most importantly, the relatively good transfer of pharmacological effects between zebrafish and humans. In the first step of the in vivo experiments, a maximum tolerated concentration for the investigated extract was identified without any apparent adverse effects such as malformations or lethality on the *Danio rerio* larvae. The viability and deformity rate of the embryos were determined after exposure to the E3 medium (control group) at serial dilutions of the extract at concentrations of 0.5, 1, 2.5, 5, 7.5, 10, 15, 25, 50 μg/mL.

**Figure 1:**
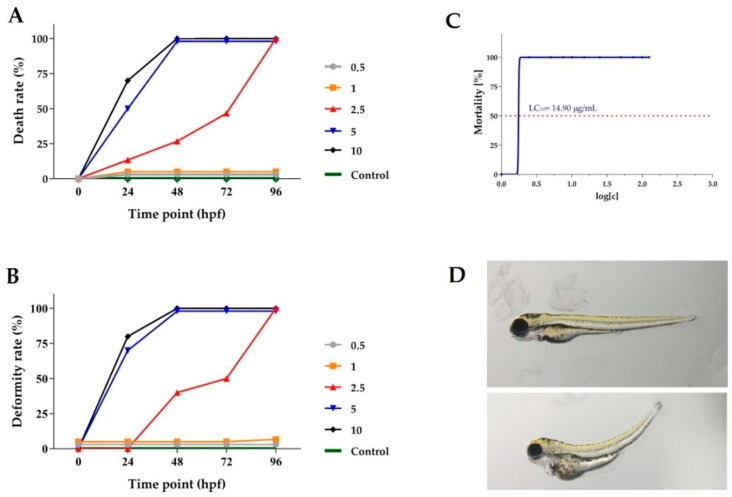
Toxicological analysis of *C.majus* extract (concentration expressed in μg/mL). (**A**) Time-response curves of zebrafish embryos death rate during incubation with a dilution series of the *C.majus* extract. Embryos exposed to concentrations above 10 μg/mL had 100% mortality. (**B**) Time-response curves of deformity rate at tested doses. (**C**) Mortality of *D. rerio* larvae in a concentration-dependent manner following *C. majus* extract exposure. The median lethal concentration (LC_50_) was based on cumulative mortality obtained from three independent experiments at 96 h post fertilization (hpf). (**D**) Representative pictures of 96 hpf zebrafish exposed to 1 μg/mL (upper picture) and 2.5 μg/mL (lower picture) of *C. majus* extract.

The exposure of the *Danio rerio* larvae to concentrations of 2.5, 5 and 10 µg/mL of the *Chelidonium majus* root extract for 96 h post fertilization (hpf) caused a 100 % mortality rate in the investigated group of larvae (Figure 1A). The exposure of the *Danio rerio* larvae to lower concentrations of the tested extract did not affect the mortality of the embryos and larvae compared to the control group. The dependence of the deformity of *Danio rerio* larvae, in a concentration-dependent manner, on *Chelidonium majus* extract exposure was presented in Figure 1B. Deformities in *Danio rerio* larvae were also observed after their exposure to the extract at higher concentrations. After 96 h of treatment with the investigated extract at concentrations of 1 μg/mL, fewer deformities compared to the control group were not observed. The median lethal concentration (LC_50_) based on the cumulative mortality obtained from three independent experiments at 96 hpf was 14.90 μg/mL (Figure 1C). Representative pictures of *Danio rerio* larvae exposed to 1 μg/mL and 2.5 μg/mL of *Chelidonium majus* extract at 96 hpf are presented in Figure 1D.

### 2.9. In Vivo Investigations of Antitumor Activity of the Chelidonium majus Root Extract

**Figure 2:**
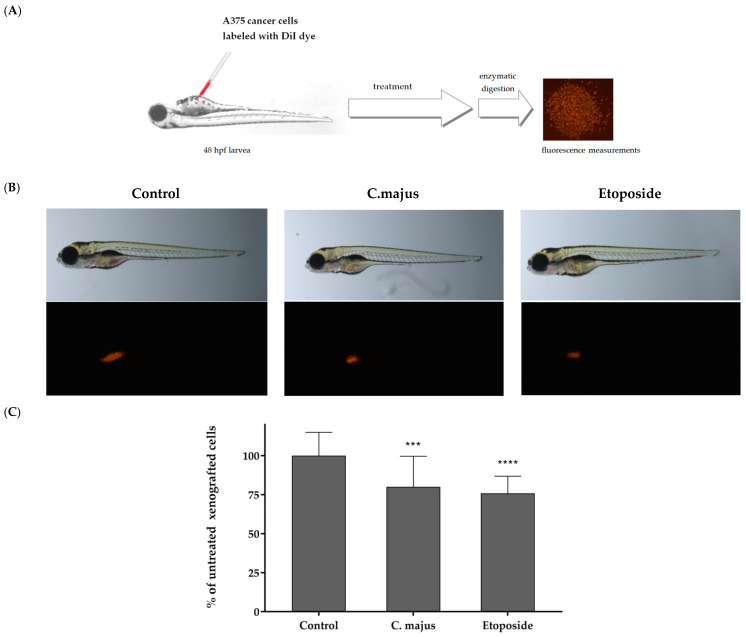
Antitumor activity of *C. majus* extract in vivo. (**A**) Schematic diagram of the experiment based on the zebrafish human tumor xenograft model. (**B**) Images of 96 hpf (hours post fertilization)/48 hpi (hours post injection) larvae. Zebrafish larvae were xenografted with A375 cells (an average of 500 cells) and treated with 0.5 μg/mL of *C. majus* extract, 5 μg/mL of reference drug (etoposide) or fish medium E3 as a control (10 larvae/group). (**C**) Inhibition of cancer cell proliferation in vivo. *** *p* < 0.001 (vs. control group; one-way ANOVA), **** *p* < 0.0001 (vs. control group; one-way ANOVA).

The *Danio rerio* larvae were xenografted with A375 cells (an average of 500 cells) and treated with 0.5 μg/mL of the *Chelidonium majus* extract or with 5 μg/mL of the reference drug etoposide or fish medium E3 (the control group). The A375 cancer cells were injected into the center of the yolk sac for the determination of the investigated extract’s anticancer activity. In Figure 2A, a scheme of the *Danio rerio* larvae xenograft experiments is presented. Figure 2B presents the results obtained for the *Danio rerio* larvae with cancer cells without any treatment (the control group), the *Danio rerio* larvae treated with the *Chelidonium majus* extract and the *Danio rerio* larvae treated with the anticancer drug etoposide. The results obtained in our experiments showed a great reduction in the number of cancer cells in the *Danio rerio* organisms after their exposure to the *Chelidonium majus* extract. The effect was similar to that obtained after treatment with etoposide (Figure 2B,C).

## 3. Materials and Methods

### 3.1. Experimental Procedure

#### 3.1.1. Chemicals and Plant Materials

Acetonitrile (ACN), methanol (MeOH, and 1-butyl-3-methylimidazolium tetrafluoroborate of chromatographic quality were received from E. Merck (Darmstadt, Germany). Dimethyl sulfoxide (DMSO) was from Sigma-Aldrich (Saint Louis, MO, USA).

Alkaloid standards (sanguinarine, chelerythrine, magnoflorine, palmatine, protopine and chelidonine) were purchased from Chem Faces Biochemical Co., Ltd. (Wuhan, China). Berberine was purchased from Sigma-Aldrich (St. Louis, MO, USA).

Plant materials were collected and identified in the Botanical Garden of Maria Curie-Skłodowska University in Lublin (Poland). Voucher specimens no. 12/2021 are available in the collection of the Department of Inorganic Chemistry, Medical University, Lublin. *Sanguinaria canadensis* was collected before flowering in March, during flowering in April and after flowering in June 2020. *Mahonia aquifolium* was collected during flowering in April. *Chelidonium majus* was collected before flowering in April, during flowering in May and after flowering in June 2018 and 2020. Plant organs were cut into pieces and dried at ambient temperature for 2 weeks.

#### 3.1.2. Apparatus and HPLC-DAD Conditions

Chromatographic analysis was performed using an LC-20AD Shimadzu (Shimadzu Corporation, Canby, OR, USA) liquid chromatograph. The chromatograph was equipped with a Shimadzu 364 SPD-M20A detector (Shimadzu Corporation, Canby, OR, USA). The DAD detector was set in the 200–800 nm range. Detection was carried out at a wavelength of 240 nm. The column oven temperature was maintained at 22 °C. An eluent flow rate of 1.0 mL/min was used. Extracts were injected into the columns using the Rheodyne 20 µL injector. Data acquisition and processing were carried out with LabSolutions software version 5.71 (Shimadzu Corporation, Kyoto, Japan). Analysis was performed on Synergi Polar RP 80A column (150 × 4.6 mm, 5 µm). The mobile phase was composed of 0.05 ML^−1^ 1-butyl-3-methylimidazolium tetrafluoroborate in water (solvent A) and 0.05 ML^−1^ 1-butyl-3-methylimidazolium tetrafluoroborate in acetonitrile (solvent B) in gradient elution: 0–10 min, 30% B; 10–20 min, 30–45% B; 20–30 min, 45% B. Calibration curves were constructed by analyzing the alkaloid standards at eight concentrations, ranging from 0.001 to 0.2 mg/mL. The calibration curves were obtained by means of the least-square method. The limit of detection (LOD) and limit of quantification (LOQ) obtained for alkaloids were calculated according to the formulas LOD = 3.3 (SD/S) and LOQ = 10 (SD/S), where SD is the standard deviation of response (peak area) and S is the slope of the calibration curve. All HPLC analyses were repeated three times.

#### 3.1.3. HPLC-MS/MS

Determination and identification of studied alkaloids were carried out using an HPLC Agilent 1290 Series system (Agilent Technologies, Germany) equipped with an ESI interface, a 6540 UHD accurate mass Q-TOF detector and Mass Hunter software version B.04.01 for data collection and instrumental control. Chromatographic XDB-C18 column (4.6 mm × 50 mm, 1.8 µm, Agilent Technologies, Waldbronn, Germany) was maintained at 20 ± 0.5 °C. The injected sample volume was 10 µL, while the mobile phase was composed of acetonitrile and 0.1% HCOOH (30:70, *v/v*) dosed at a flow rate of 0.6 mL/min. Addition of 0.1% formic acid to the acetonitrile/water mobile phase could improve peak shapes and increase MS detection sensitivity. The Q-TOF-MS parameters were optimized with the use of Box–Behnken approach (*Design of Experiments*). 

Quadrupole time-of-flight mass spectrometric analyses were performed using electrospray ion source operating in positive ion mode (ESI(+)), with the following set of operation parameters: capillary voltage (CV), 3.5 kV; octopole voltage (OV), 800 V; skimmer voltage (SV), 50 V; drying gas temperature (DGT), 250 °C; shielding gas temperature (SGT), 315 °C; fragmentor voltage (FV), 195 V. An optimization study for the three most important parameters (DGT (*X*_1_), FV (*X*_2_) and CV (*X*_3_)) was performed with the use of Statistica 12.0. The whole Box–Behnken design consisted of 15 factorial points.

The Q-TOF and information-dependent acquisition scan were operated with a mass range of 40 to 370 *m*/*z*. Nitrogen was used as drying (7 L/min) and nebulizing (40 psig) gas. High-purity nitrogen gas was used for the nebulizer/DuosprayTM and curtain gases. The instrument was operated in extracted ion chromatogram (EIC) and product ion modes, respectively. Product ion calibration was performed in both high-sensitivity and high-resolution modes using a calibrant delivery system before analysis. The HPLC-MS/MS data acquisition and processing were carried out using MassHunter Workstation software. The data were further processed using Microsoft Excel.

#### 3.1.4. Extraction Procedure

The previously described procedure of alkaloids extraction from plant materials was applied after minor modifications [32]. Weighted samples (5 g) of each plant material were macerated with 100 mL of ethanol for 72 h. In the next step, samples were extracted in an ultrasonic bath for 5 h at ambient temperature. Obtained extracts were filtered, the solvent evaporated under vacuum, and the residues dissolved in 30 mL of 2% sulfuric acid. Next, samples were defatted with diethyl ether (3 × 40 mL). Subsequently, the aqueous layers were basified with 25% ammonia to a pH of 9.5–10, and the alkaloids extracted with chloroform (3 × 50 mL). After evaporation of the organic solvent, the dried extracts were dissolved in 5 mL of MeOH.

#### 3.1.5. Investigation of Cytotoxic Activity

##### Cell Cultivation

Cytotoxic activity of the investigated plant extracts was evaluated using the following cancer cell lines: FaDu (human pharyngeal squamous carcinoma cells), SCC-25 (human tongue squamous carcinoma cells), MCF-7 (human breast adenocarcinoma cells), MDA-MB-231 (human triple-negative breast adenocarcinoma cells), A375, G361 and SK-MEL-3 (malignant melanoma cells). Moreover, human normal skin fibroblasts (CRL-1634) were used as control cells. The cell lines were obtained from American Type Culture Collection (ATCC; Manassas, VA, USA). MCF-7, MDA-MB-231, A375 and CRL-1634 cells were cultured using Dulbecco’s Modified Eagle’s Medium—high glucose (DMEM) containing 10% fetal bovine serum, 100 U/mL of penicillin and 100 mg/mL of streptomycin. FaDu cells were cultured using Eagle’s Minimum Essential Medium (MEM) supplemented with 10% fetal bovine serum, 100 U/mL of penicillin and 100 mg/mL of streptomycin. SCC-25 cell line was cultured in Dulbecco’s Modified Eagle’s Medium/Nutrient Mixture F-12 Ham (DMEM/F12) supplemented with 10% fetal bovine serum, 400 ng/mL hydrocortisone, 100 U/mL of penicillin and 100 mg/mL of streptomycin (all from Sigma Aldrich). Human melanoma SK-MEL-3 and G-361 cells were maintained in McCoy’s 5A Medium (Sigma Aldrich, St. Louis, MO, USA) supplemented with 15% (for SK-MEL-3) or 10% (for G-361) FBS, penicillin (100 U/mL) and streptomycin (100 μg/mL). The cells were maintained in a 5% CO_2_ atmosphere at 37 °C. 

##### MTT Assay

The dried plant extracts were dissolved in DMSO in order to obtain stock solutions (50 mg/mL). On the day of the experiment, the suspension of cells (1 × 105 cells/mL) in the respective medium was applied to a 96-well plate at 100 μL per well. Cells were incubated for 24 h, and subsequently, the medium was removed from the wells and replaced by increasing concentrations of plant extracts or reference drugs dissolved in medium containing 2% FBS. Possible cytotoxicity of DMSO was also examined at concentrations used in respective dilutions of stock solutions. After 24 h incubation, 15 μL of MTT working solution (5 mg/mL in PBS) was added to each well. The plate was incubated for 3 h. Subsequently, 100 μL of 10% SDS solution was added to each well. Cells were incubated overnight at 37 °C to dissolve the precipitated formazan crystals. The concentration of the dissolved formazan was determined by measuring the absorbance at μL = 570 nm using a microplate reader (Epoch, BioTek Instruments, Inc., Winooski, VT, USA). The mean results of cell viability were obtained from two independent experiments performed in triplicate. DMSO in the concentrations present in the dilutions of stock solutions did not influence the viability of the tested cells. IC50 values of the investigated extracts were calculated using IC50 online calculator (www.IC50.tk, accessed on 15 December 2022).

##### BrdU Assay

The antiproliferative effect of the selected extracts was measured by means of the BrdU assay, which indicates the rate of incorporation of 5′-bromo-2′-deoxy-uridine (BrdU) during DNA biosynthesis. In brief, cancer cells were plated in 96-well plates (NUNC, Roskilde, Denmark) at a density of 5 × 104 cells/mL. The next day, cells were exposed to increased concentrations of the extracts, reference drugs or a fresh culture medium. Cell proliferation was examined after 24 h incubation, according to the manufacturer’s protocol (Cell Proliferation ELISA BrdU, Roche Diagnostics GmbH, Penzberg, Germany). Antiproliferative effect of the investigated extracts and reference drugs was expressed as IC50 values, calculated using IC50 online calculator (www.IC50.tk, accessed on 15 December 2022).

#### 3.1.6. Danio Rerio Culture and Fish Embryo Toxicity Test (FET)

*Danio rerio* larvae of the AB strain (Experimental Medicine Centre, Medical University of Lublin, Poland) were maintained at 28 ± 0.5 °C on a 14/10 h light/dark cycle under standard aquaculture conditions. After mating, the fertilized eggs were collected within 30 min. Embryos were reared in E3 embryo medium (pH 7.1–7.3; 17.4 µM NaCl, 0.21 µM KCl, 0.12 µM MgSO_4_ and 0.18 µM Ca(NO_3_)_2_) in an incubator (IN 110 Memmert GmbH, Germany) at 28 ± 0.5 °C. Zebrafish embryos were examined to remove unfertilized, coagulated and damaged samples. The FET test was performed based on OECD Guidelines for the Testing of Chemicals, Test No. 236. The examined extract was weighted, dissolved in DMSO as stock solution and diluted in E3 embryo medium to the indicated treatment concentrations. Stock and dilutions in E3 embryo medium were prepared freshly before testing. Embryos were exposed to E3 medium (control group) or serial dilutions of the *Chelidonium majus* root extract (0.5, 1, 2.5, 5, 7.5, 10, 15, 25 and 50 μg/mL). The final DMSO concentration had no detectable effects on zebrafish development. The test was conducted in 24-well plates with 5 embryos per well and 10 per group, in triplicate. The covered plates were kept at 28 ± 0.5 °C under light/dark conditions (12 h/12 h). Embryonic viability and malformation rates of each treatment group were recorded at 24, 48, 72 and 96 hpf. All experiments were conducted in accordance with the National Institutes of Health Guidelines for the Care and Use of Laboratory Animals and the European Community Council Directive for the Care and Use of Laboratory Animals (2010/63/EU). For the experiment with larvae up to 5 dpf, the agreement of the Local Ethical Commission is not required.

#### 3.1.7. Danio Rerio Human Tumor Cell Xenograft

Danio rerio embryos were obtained using standard mating conditions. Before xenoplantation at 48 hpf, embryos were dechorionized using microforceps, anesthetized with 0.0016% tricaine and positioned on their left side on a wet Petri dish with microscope slide. A375 cells were detached from culture dishes using 0.25% Trypsin-EDTA and washed twice with PBS at room temperature. Cells were stained with 5 μM DiI diluted in PBS for 20 min at 37 °C and washed three times with PBS (according to manufacturer instructions). Cancer cells were counted by microscopy and injected into the center of the yolk sac using a microinjector (NARISHIGE, IM-300, Tokyo, Japan) and micromanipulator (World Precision Instruments, 3301R, Sarasota, FL, USA) equipped with borosilicate glass capillaries (World Precision Instruments, Sarasota, FL, USA). After injection, embryos were transferred into 96-well plates and incubated at 0.5 μg/mL concentration of the extract diluted in E3 media. The control group consisted of injected embryos in E3 medium. Injected embryos were maintained at 32 °C for 3 days and analyzed for cancer cell proliferation. After 3 days post injection (dpi), larvae were anesthetized with 0.0016% tricaine and digested, according to the procedure described previously [33], in order to obtain the single-cell solution. An 8% paraformaldehyde solution was used for fixation of the cells. Images were captured with a ZEISS SteREO Discovery.V8 microscope and Zen 2.3 lite software (Carl Zeiss Microscopy GmbH, Jena, Germany). The cells were counted using ImageJ software version 1.53t. For statistical analysis, GraphPad Prism 5.0 was used (GraphPad Software Inc., La Jolla, CA, USA). Statistical differences between the investigated groups (control group, etoposide-treated group and Chelidonium majus extract-treated group) were analyzed using one-way ANOVA with a post hoc Tukey test.

## 4. Conclusions

The investigated plant extracts obtained from different parts of *Chelidonium majus, Mahonia aquifolium* and *Sanguinaria canadensis* collected at various vegetation stages and containing high concentrations of isoquinoline alkaloids exhibited anticancer activity. The highest activity against all the tested cancer cell lines was found for sanguinarine and chelerythrine.

The obtained data demonstrated that the extracts obtained from *Chelidonium majus, Mahonia aquifolium* and *Sanguinaria canadensis* exhibit very high cytotoxic activity against the MCF-7, MDA-MB-231, SCC-25 and FaDu cancer cell lines. 

To the best of our knowledge, the cytotoxic activity of these extracts as regards the time of plant material collection has not been previously investigated against the cell lines tested in our experiments. The cytotoxicity of the *Sanguinaria candensis* extracts on the MCF-7, SCC-25 and FaDu cell lines and the cytotoxicity of the *Chelidonium majus* extracts on the G361 and SK-MEL-3 cell lines have also been investigated for the first time.

Almost all the investigated plant extracts exhibited higher cytotoxicity than the cytotoxic activity of the anticancer drug etoposide, which evidently indicated the very high cytotoxic potential of the investigated alkaloids and plant extracts against the tested cancer cells. The highest cytotoxicity was found for the extracts obtained from the whole *Sanguinaria canadensis* plant and the *Chelidonium majus* root, which contain highly cytotoxic alkaloids: sanguinarine and chelerythrine. 

Differences were also observed in the cytotoxic activity of the extracts obtained from various parts of the investigated plants. The highest cytotoxicity was exhibited by the extracts obtained from the *Chelidonium majus* root in May and June against the MCF-7 cell line (IC_50_ = 0.17 μg/mL), MDA-MB-231 cells (IC_50_ = 0.32 μg/mL) and SCC-25 cells (IC_50_ = 0.62 μg/mL). The lowest IC_50_ (0.14 μg/mL) value on the FaDu cells was obtained after treatment with the extract obtained from the *Chelidonium majus* herb collected in May.

Our data demonstrated that the extracts obtained from different parts of *Chelidonium majus,* especially from the plant’s herb and root, also displayed high cytotoxic activity against the A375, G-361 and SK-MEL-3 melanoma cell lines. 

The highest cytotoxicity against the A375 cell line was observed after exposure to the extract obtained from the *Chelidonium majus* root, with an IC_50_ of 12.65 μg/mL. All the investigated extracts were highly cytotoxic against G361 cells, with IC_50_ values ranging from 2.27 μg/mL after treatment with the herb extract to 6.97 μg/mL when the cells were exposed to the extract obtained from seeds. The lowest viability of the SK-MEL-3 cells was determined after exposure to the *Chelidonium majus* root extract (IC_50_ = 1.93 μg/mL). The extract obtained from the *Chelidonium majus* seeds exhibited the lowest cytotoxic activity, especially against MCF-7, MDA-MB-231 and A375 cancer cells. Therefore, it is not recommended for further investigation in terms of its activity.

Most of the investigated *Chelidonium majus* extracts exhibited higher cytotoxicity than the cytotoxic activity of the anticancer drugs etoposide, cisplatin and hydroxyurea, which clearly indicates a very high cytotoxicity potential against the tested melanoma cells of the plant extracts. 

In vivo experiments performed for the first time using a Danio rerio larvae xenograft model for the determination of the cytotoxicity of the *Chelidonium majus* root extract confirmed a great effect of the extract on the decrease in the number of cancer cells in a living organism. Based on our results, all the other investigated plant extracts, especially those obtained from the *Chelidonium majus* root and the whole *Sanguinaria canadensis* plant, can be recommended for further in vivo experiments. The investigated extracts, especially those obtained from the *Chelidonium majus* root, exhibited in vivo cytotoxic activity, and the alkaloids contained therein may be developed as new candidate anticancer agents.

## Figures and Tables

**Table 1:** Contents of isoquinoline alkaloids in plant extracts (mg/g of dry plant material).

Plant Extract	Berberine	Chelerythrine	Chelidonine	Magnoflorine	Palmatine	Protopine	Sanguinarine
*Chelidonium majus* herb before flowering	0.168	0.024	0.891	-	-	0.984	0.075
*Chelidonium majus* root before flowering	0.184	1.105	1.341	-	-	0.358	0.344
*Chelidonium majus* herb during flowering	0.120	0.037	0.317	-	-	0.690	0.081
*Chelidonium majus* root during flowering	0.023	1.182	0.967	-	-	0.559	0.638
*Chelidonium majus* herb after flowering	0.148	0.130	0.479	-	-	1.227	0.128
*Chelidonium majus* root after flowering	0.011	1.744	1.490	-	-	0.308	0.821
*Chelidonium majus* seeds after flowering	0.017	0.002	0.069	-	-	0.008	0.003
*Chelidonium majus* pods after flowering	0.174	0.245	0.545	-	-	0.133	0.545
*Mahonia aquifolium* cortex before flowering	0.135	-	-	0.087	0.039	-	-
*Mahonia aquifolium* cortex during flowering	3.316	-	-	-	0.794	-	-
*Mahonia aquifolium* cortex after flowering	0.067	-	-	0.102	0.793	-	-
*Mahonia aquifolium* leaves before flowering	-	-	-	0.324	-	-	-
*Mahonia aquifolium* leaves during flowering	0.0220	-	-	0.238	<LOQ	-	-
*Mahonia aquifolium* leaves after flowering	<LOQ	-	-	0.319	-	-	-
*Mahonia aquifolium* barked stalk during flowering	0.438	-	-	-	0.045	-	-
*Mahonia aquifolium* barked stalk after flowering	0.044	-	-	0.191	0.342	-	-
*Mahonia aquifolium* root during flowering	7.046	-	-	-	0.629	-	-
*Mahonia aquifolium* root after flowering	0.008	-	-	-	0.305	-	-
*Sanguinaria canadensis* before flowering	0.006	2.738	-	-	<LOQ	-	4.874
*Sanguinaria canadensis* during flowering	0.014	5.362	<LOQ	-	-	0.015	9.598
*Sanguinaria canadensis* after flowering	0.010	6.878	-	-	-	0.110	6.949

LOQ—value below limit of quantification; —peak not identified. Berberine has been identified in all investigated plants, chelerythrine in *Chelidonium majus* and *Sanguinaria canadensis*, chelidonine in all investigated *Chelidonium majus* extracts and, though in very low concentration, in *Sanguinaria canadensis* extract collected during flowering. The presence of magnoflorine was found in the extracts obtained from various parts of *Mahonia aquifolium*. Palmatine was also detected in *Mahonia aquifolium* extracts. Protopine was determined in all extracts from *Chelidonium majus* and in small amounts in the extracts obtained from *Sanguinaria canadensis* collected both during and after flowering. Sanguinarine was identified in all extracts obtained from *Chelidonium majus* and *Sanguinaria canadensis.*

**Table 2:** Retention time (t_R_) and asymmetry factor (As) obtained by HPLC-DAD and MS parameters and conditions used for determination and identification of selected alkaloids in plant extract samples.

Compound	HPLC-DAD		LC-MS									
	t_R_	As	Elemental Composition	t_R_ (min)	Polarity	Theoretical (*m*/*z*)	Measured (*m*/*z*)	Major Fragment Ions	Error (ppm)	ID Score [%]	Collision Energy (eV)	Fragmentor Voltage
Berberine	19.70	1.05	C_20_H_18_NO_4_ [M+H]^+^	3.12	ESI+	335.7426	335.7429	319.7029 305.6823 304.1893 291.6987 277.6827	−0.41	99.18	20	195
Chelerythrine	23.32	1.06	C_21_H_18_NO_4_ [M+H]^+^	3.98	ESI+	347.7491	347.7489	331.7071 303.6990 274.6920 231.6968	1.19	99.56	20	195
Chelidonine	10.91	1.07	C_20_H_20_NO_5_ [M+H]^+^	1.94	ESI+	354.3922	354.3920	336.6503 303.6990 274.6920 189.4355	−1.17	99.90	20	195
Magnoflorine	3.94	1.08	C_20_H_24_NO_4_ [M+H]^+^	3.27	ESI+	341.7915	341.7917	296.7147 264.6899 236.7089 206.7327	−0.39	99.48	20	195
Palmatine	16.96	1.18	C_21_H_22_NO_4_ [M+H]^+^	2.53	ESI+	351.7872	351.7853	335.7442 307.7351 277.6835 249.6977	1.07	99.62	20	195
Protopine	8.49	1.03	C_20_H_20_NO_5_ [M+H]^+^	1.68	ESI+	353.7653	353.7655	336.1209 274.6716 205.7380 188.7681 148.8711	−0.93	99.15	20	195
Sanguinarine	20.37	1.06	C_20_H_14_NO_4_ [M+H]^+^	2.75	ESI+	331.7068	331.7065	316.6761 303.6993 288.6723 273.6853 245.7012	1.26	99.34	20	195

**Table 3:** Cytotoxic effect of the investigated plant extracts against cancer cell lines (FaDu, SCC-25, MCF-7 and MDA-MB-231) expressed as IC_50_ in μg/mL and ±SD values.

Plant Extract	IC_50_ [μg/mL] ± SD			
	MCF-7	MDA-MB-231	SCC-25	FaDu
*Chelidonium majus* herb after flowering	17.20 ± 2.07	25.31 ± 2.41	1.76 ± 0.29	3.68 ± 0.63
*Chelidonium majus* root after flowering	7.72 ± 0.28	17.01 ± 1.68	0.95 ± 0.02	2.06 ± 0.17
*Chelidonium majus* seeds after flowering	>100	>100	13.74 ± 1.17	18.38 ± 1.78
*Chelidonium majus* pods after flowering	8.26 ± 1.07	>100	5.05 ± 0.36	3.94 ± 0.33
*Mahonia aquifolium* root during flowering	34.79 ± 3.29	24.00 ± 2.63	36.98 ± 4.28	10.85 ± 1.86
*Mahonia aquifolium* barked stalk during flowering	29.48 ± 2.38	89.70 ± 6.03	28.44 ± 1.36	5.73 ± 1.07
*Mahonia aquifalium* leaves during flowering *	89.14 ± 2.73	90.71 ± 7.29	97.25 ± 8.07	46.77 ± 7.84
*Mahonia aquifalium* cortex during flowering *	15.71 ± 1.92	31.87 ± 4.35	31.37 ± 2.29	7.67 ± 0.82
*Sanguinaria candensis* before flowering	1.48 ± 0.17	4.15 ± 0.39	0.90 ± 0.03	0.21 ± 0.02
*Sanguinaria candensis* during flowering	1.36 ± 0.11	4.26 ± 0.43	2.99 ± 0.23	0.27 ± 0.04
*Sanguinaria candensis* after flowering	3.14 ± 0.62	11.37 ± 0.77	1.05 ± 0.08	0.41 ± 0.09
Etoposide	136.48 ± 8.95	219.31 ± 24.47	223.94 ± 24.81	38.73 ± 1.56

* Data previously published [32].

**Table 4:** Cytotoxic effect of extracts from *Chelidonium majus* collected in various vegetation steps against cancer cell lines (FaDu, SCC-25, MCF-7 and MDA-MB-231) and fibroblasts expressed as IC_50_ in μg/mL and ±SD values.

Plant Extract	IC_50_ [μg/mL] ± SD				
	MCF-7	MDA-MB-231	SCC-25	FaDu	Fibroblasts
*Chelidonium majus* root 10.04.2018	5.46 ± 0.01	5.77 ± 0.37	1.26 ± 0.23	3.67 ± 0.21	0.62 ± 0.12 (0.96 ± 0.12)
*Chelidonium majus* root 10.05.2018	0.20 ± 0.03 (0.18 ± 0.02)	0.32 ± 0.04 (0.24 ± 0.04)	0.62 ± 0.10 (0.41 ± 0.03)	1.96 ± 0.17	0.67 ± 0.06 (0.85 ± 0.05)
*Chelidonium majus* root 10.06.2018	0.17 ± 0.02 (0.11 ± 0.01)	1.49 ± 0.36	0.83 ± 0.09	2.56 ± 0.47	0.76 ± 0.10 (0.68 ± 0.06)
*Chelidonium majus* herb 10.04.2018	28.23 ± 3.67	43.70 ± 4.79	75.62 ± 4.28	13.10 ± 1.50 (8.94 ± 0.47)	16.58 ± 1.27 (17.52 ± 0.71)
*Chelidonium majus* herb 10.05.2018	28.03 ± 2.04	9.36 ± 0.77	32.52 ± 5.01	0.14 ± 0.05 (0.10 ± 0.01)	0.56 ± 0.03 (0.85 ± 0.10)
*Chelidonium Majus* herb 10.06.2018	11.97 ± 0.30 (8.03 ± 0.37)	24.68 ± 2.45	26.93 ± 3.12	18.34 ± 2.17 (14.28 ± 0.77)	18.78 ± 0.58 (20.79 ± 1.04)

Underlined—IC_50_ obtained for cancer cells lower than those obtained for fibroblasts. The values obtained by the BrdU test are given in parentheses.

**Table 5:** Cytotoxic effect of extracts from various parts of *Chelidonium majus* collected after flowering and anticancer drugs against melanoma cell lines (A375, G-361 and SK-MEL-3) expressed as IC_50_ in μg/mL and ±SD values.

Plant Extract	IC_50_ [μg/mL] ± SD		
	A375	G361	SK-MEL-3
*Chelidonium majus* herb	27.91 ± 2.76 (18.08 ± 1.32)	2.27 ± 0.32 (4.16 ± 0.51)	7.89 ± 0.57 (3.62 ± 0.13)
*Chelidonium majus* root	12.65 ± 1.85 (7.24 ± 0.21)	4.17 ± 0.29 (3.98 ± 0.14)	1.93 ± 0.08 (1.17 ± 0.07)
*Chelidonium majus* seeds	>200 (142.65 ± 4.65)	6.97 ± 0.75 (5.17 ± 0.24)	33.33 ± 4.70 (19.73 ± 0.45)
*Chelidonium majus* pods with seeds	180.16 ± 12.51 (120.47 ± 4.83)	5.77 ± 0.43 (4.19 ± 0.18)	19.85 ± 2.07 (11.05 ± 0.81)
Etoposide	92.34 ± 4.58 (32.8 ± 2.38)	52.32 ± 3.86 (18.01 ± 0.57)	>200 (>100)
Cisplatin	10.62 ± 1.04 (6.02 ± 0.41)	11.53 ± 1.46 (4.85 ±0.33)	14.42 ± 1.61 (7.58 ± 0.73)
Hydroxyurea	>200	>200	>200

The values obtained by the BrdU test are given in parentheses.

**Table 6:** Equation of correlation curve and correlation coefficients (r); values between isoquinoline alkaloid contents and cytotoxic activity expressed as IC_50_ of investigated plant extracts.

Cell Line	*Chelerythrine*		*Sanguinarine*		*Chelerythrine and* *sanguinarine*	
	Equation	r	Equation	r	Equation	r
*MCF-7*	y = −15.72x + 23.26	0.8845	y = −35.25x + 24.60	0.8608	y = −11.02x + 23.83	0.8835
*MDA-MB-231*	y = −17.11x + 26.10	0.7391	y = −37.98x + 27.43	0.7120	y = −11.97x + 26.69	0.7360
*SCC-25*	y = −32.13x + 45.28	0.7964	y = −69.04x + 46.98	0.7426	y = −22.26x + 46.16	0.7856
*FaDu*	y = −5.357x + 10.35	0.5340	y = −11.71x + 10.70	0.5065	y = −3.729x + 10.52	0.5293

## Data Availability

Not applicable.

## Supplementary Materials

The following supporting information can be downloaded at: https://www.mdpi.com/article/10.3390/ijms24076360/s1.

## References

[1] Hammerova J., Uldrijan S., Taborska E., Slaninova I. (2011). Benzo[c]phenanthridine alkaloids exhibit strong anti-proliferative activity in malignant melanoma cells regardless of their p53 status. J. Dermatol. Sci..

[2] Yun D., Yoon S.Y., Park S.J., Park Y.J. (2021). The Anticancer Effect of Natural Plant Alkaloid Isoquinolines. Int. J. Mol. Sci..

[3] Warowicka A., Popenda Ł., Bartkowiak G., Musidlak O., Litowczenko-Cybulska J., Kuźma D., Nawrot R., Jurga S., Goździcka-Józefiak A. (2019). Protoberberine compounds extracted from *Chelidonium majus* L. as novel natural photosensitizers for cancer therapy. Phytomedicine.

[4] Kulp M., Bragina O. (2013). Capillary electrophoretic study of the synergistic biological effects of alkaloids from *Chelidonium majus* L. in normal and cancer cells. Anal. Bioanal. Chem..

[5] El-Readi M.Z., Eid S.Y., Ashour M.L., Tahrani A., Wink M. (2013). Modulation of multidrug resistance in cancer cells by chelidonine and *Chelidonium majus* alkaloids. Phytomedicine.

[6] Wu C., Wang X., Xu M., Liu Y., Di X. (2019). Intracellular Accumulation as an Indicator of Cytotoxicity to Screen Hepatotoxic Components of *Chelidonium majus* L. by LC–MS/MS. Molecules.

[7] Capistrano I.R., Wouters A., Lardon F., Gravekamp C., Apers S., Pieters L. (2015). In Vitro and in vivo investigations on the antitumour activity of *Chelidonium majus*. Phytomedicine.

[8] Gu Y., Qian D., Duan J.-a., Wang Z., Guo J., Tang Y., Guo S. (2010). Simultaneous determination of seven main alkaloids of *Chelidonium majus* L. by ultraperformance LC with photodiode-array detection. J. Sep. Sci..

[9] Huang Y., Wang T., Yin G., Wang J., Jiang K., Tu J. (2020). High-performance liquid chromatography–based fingerprint analysis with chemical pattern recognition for evaluation of *Mahonia bealei* (Fort.). Carr. J. Sep. Sci..

[10] Wang W., Ma X., Guo X., Zhao M., Tu P., Jiang Y. (2015). A series of strategies for solving the shortage of reference standardsfor multi-components determination of traditional Chinese medicine, *Mahoniae* Caulis as a case. J. Chromatogr. A.

[11] Singh A., Bajpai V., Kumar S., Rawat A.K.S., Kumar B. (2017). Analysis of isoquinoline alkaloids from *Mahonia leschenaultia* and *Mahonia napaulensis* roots using UHPLC-Orbitrap-MSn and UHPLC-QqQLIT-MS/MS. J. Pharm. Anal..

[12] Park S.-W., Kim S.R., Kim Y., Lee J.-H., Woo H.-J., Yoon Y.-K., Kim Y.I. (2015). *Chelidonium majus* L. extract induces apoptosis through caspase activity via MAPK-independent NF-κB signalling in human epidermoid carcinoma A431 cells. Oncol. Rep..

[13] Havelek R., Seifrtova M., Kralovec K., Krocova E., Tejkalova V., Novotny I., Cahlikova L., Safratova M., Opletal L., Bilkova Z. (2016). Comparative cytotoxicity of chelidonine and homochelidonine, the dimethoxy analogues isolated from *Chelidonium majus* L. (Papaveraceae), against human leukemic and lung carcinoma cells. Phytomedicine.

[14] Zhang Z.H., Mi C., Wang K.S., Wang Z., Li M.Y., Zuo H.X., Xu G.H., Li X., Piao L.X., Ma J. (2018). Chelidonine inhibits TNF-α-induced inflammation by suppressing the NF-κB pathways in HCT116 cells. Phytother. Res..

[15] Singh T., Vaid M., Katiyar N., Sharma S., Katiyar S.K. (2011). Berberine, an isoquinoline alkaloid, inhibits melanoma cancer cell migration by reducing the expressions of cyclooxygenase-2, prostaglandin E2 and prostaglandin E2 receptors. Carcinogenesis.

[16] Liu J.-F., Lai K.C., Peng S.-F., Maraming P., Huang Y.-P., Huang A.-C., Chueh F.-S., Huang W.-W., Chung J.-G. (2018). Berberine Inhibits Human Melanoma A375.S2 Cell Migration and Invasion via Affecting the FAK, uPA, and NF-B Signaling Pathways and Inhibits PLX4032 Resistant A375.S2 Cell Migration In Vitro. Molecules.

[17] Ren M., Yang L., Li D., Yang L., Su Y., Su X. (2020). Cell Cycle Regulation by Berberine in Human Melanoma A375 Cells. Bull. Exp. Biol. Med..

[18] Kim J.-H., Ryu A.-R., Kang M.-J., Lee M.-Y. (2016). Berberine-induced changes in protein expression and antioxidant enzymes in melanoma cells. Mol. Cell. Toxicol..

[19] Kou Y., Li L., Li H., Tan Y., Li B., Wang K., Du B. (2016). Berberine suppressed epithelial mesenchymal transition through cross-talk regulation of PI3K/AKT and RARa/RARb in melanoma cells. Biochem. Biophys. Res. Commun..

[20] He J.M., Mu Q. (2015). The medicinal uses of the genus *Mahonia* in traditional Chinese medicine: An ethnopharmacological, phytochemical and pharmacological review. J. Ethnopharmacol..

[21] Godevac D., Damjanovic A., Stanojkovic T.P., Anđelkovic B., Zdunic G. (2018). Identification of cytotoxic metabolites from *Mahonia aquifolium* using1H NMR-based metabolomics approach. J. Pharm. Biomed. Anal..

[22] Rezadoost M.H., Kumleh H.H., Ghasempour A. (2019). Cytotoxicity and apoptosis induction in breast cancer, skin cancer and glioblastoma cells by plant extracts. Mol. Biol. Rep..

[23] Damjanovic A., Kolundžija B., Matic I.Z., Krivokuca A., Zdunic G., Šavikin K., Jankovic R., Stankovic J.A., Stanojkovic T.P. (2020). *Mahonia aquifolium* Extracts Promote Doxorubicin Effects against Lung Adenocarcinoma Cells In Vitro. Molecules.

[24] Andreicuț A.D., Fischer-Fodor E., APârvu E., Ţigu A.B., Cenariu M., Pârvu M., Cătoi F.A., Irimie A. (2019). Antitumoral and Immunomodulatory Effect of *Mahonia aquifolium* Extracts. Oxid. Med. Cell. Longev..

[25] Ma W.-K., Li H., Dong C.-L., He X., Guo C.-R., Zhang C.-F., Yu C.-H., Wang C.-Z., Yuan C.-S. (2016). Palmatine from *Mahonia bealei* attenuates gut tumorigenesis in ApcMin/+ mice via inhibition of inflammatory cytokines. Mol. Med. Rep..

[26] Wong B.-S., Hsiao Y.-C., Lin T.-W., Chen K.-S., Chen P.-N., Kuo W.-H., Chu S.-C., Hsieh Y.-S. (2009). The in vitro and in vivo apoptotic effects of *Mahonia oiwakensis* on human lung cancer cells. Chem. Biol. Interact..

[27] Senchina D.S., Flinn G.N., McCann D.A., Kohut M.L., Shearn C.T. (2009). Bloodroot (*Sanguinaria canadensis* L., Papaveraceae) Enhances Proliferation and Cytokine Production by Human Peripheral Blood Mononuclear Cells in an In Vitro Model. J. Herbs Spices Med. Plants.

[28] Tuzimski T., Petruczynik A., Plech T., Kaproń B., Makuch-Kocka A., Szultka-Młyńska M., Misiurek J., Buszewski B. (2021). Determination of Cytotoxic Activity of *Sanguinaria Canadensis* Extracts against Human Melanoma Cells and Comparison of Their Cytotoxicity with Cytotoxicity of Some Anticancer Drugs. Molecules.

[29] Petruczynik A., Plech T., Tuzimski T., Misiurek J., Kaproń B., Misiurek D., Szultka-Młyńska M., Buszewski B., Waksmundzka-Hajnos M. (2019). Determination of Selected Isoquinoline Alkaloids from *Mahonia aquifolia*; *Meconopsis cambrica*; *Corydalis lutea*; *Dicentra spectabilis*; *Fumaria offcinalis*; *Macleaya cordata* Extracts by HPLC-DAD and Comparison of Their Cytotoxic Activity. Toxins.

[30] Kulp M., Bragina O., Kogerman P., Kaljurand M. (2011). Capillary electrophoresis with led-induced native fluorescence detection for determination of isoquinoline alkaloids and their cytotoxicity in extracts of *Chelidonium majus* L.. J. Chromatogr. A.

[31] Campbell S., Affolter J., Randle W. (2007). Spatial and temporal distribution of the alkaloid sanguinarine in *Sanguinaria canadensis* L. (Bloodroot). Econ. Bot..

[32] Petruczynik A., Tuzimski T., Plech T., Misiurek J., Szalast K., Szymczak G. (2019). Comparison of Anticancer Activity and HPLC-DAD Determination of Selected Isoquinoline Alkaloids from *Thalictrum foetidum*, *Berberis* sp. and *Chelidonium majus* Extracts. Molecules.

[33] Bresciani E., Broadbridge E., Liu P.P. (2018). An efficient dissociation protocol for generation of single cell suspension from zebrafish embryos and larvae. Methods X.

