# Supplementary material for: Determination of Selected Isoquinoline Alkaloids from Chelidonium majus, Mahonia aquifolium and Sanguinaria canadensis Extracts by Liquid Chromatography and Their In Vitro and In Vivo Cytotoxic Activity against Human Cancer Cells
Source: Int J Mol Sci. 2023 Mar 28;24(7):6360. doi: 10.3390/ijms24076360 (PMC10093986; doi:10.3390/ijms24076360)
Supplement: Supplementary file 1 [file ijms-24-06360-s001.zip › ijms-2260827-supplementary.pdf]

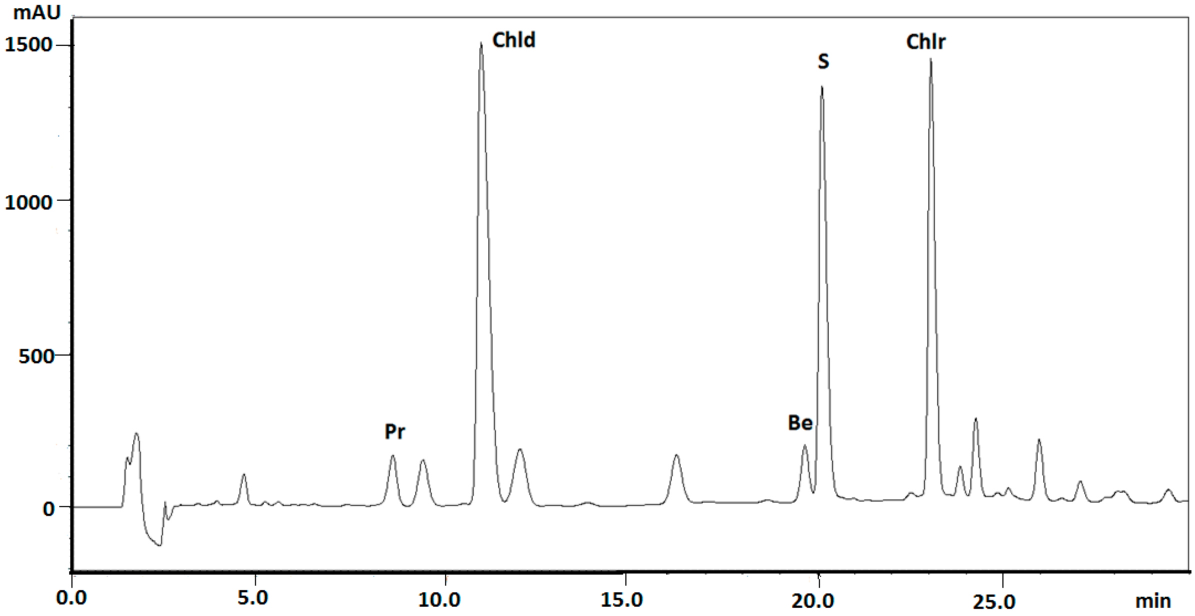

Figure S1. A. Chromatogram obtained for *Chelidonium majus* root extract from plant material collected during flowering. Abbreviations: Pr-protopine, Chld-chelidonine, Be-berberine, S-sanquinarine, Chlr-chelerythrine.

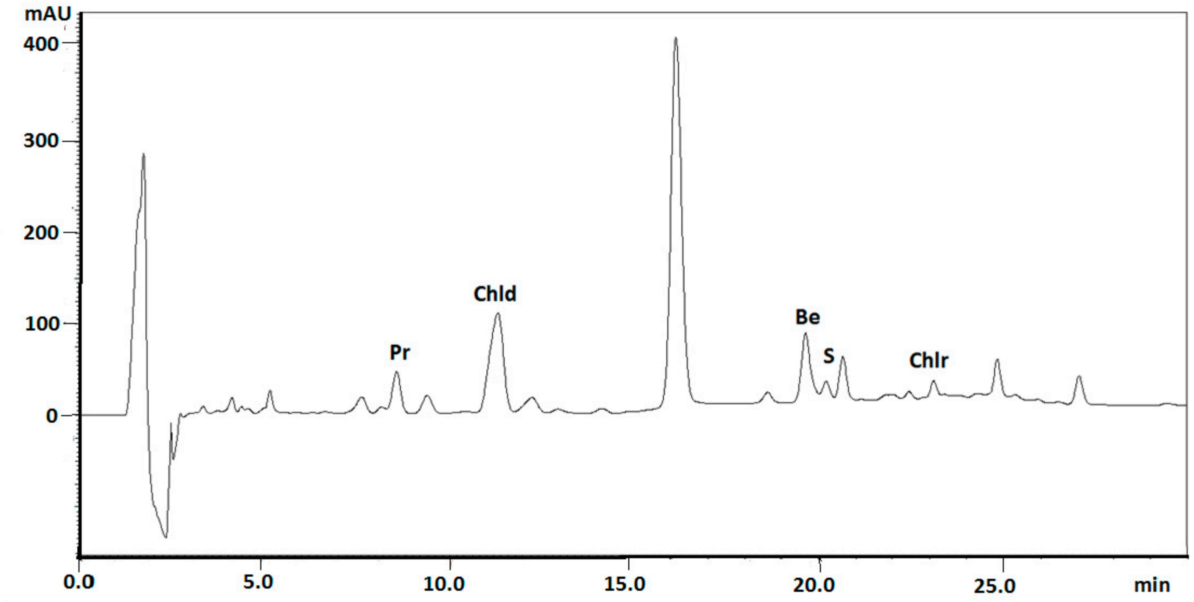

Figure S1. B. Chromatogram obtained for *Chelidonium majus* herb extract from plant material collected during flowering. Abbreviations: Pr-protopine, Chld-chelidonine, Be-berberine, S-sanquinarine, Chlr-chelerythrine.

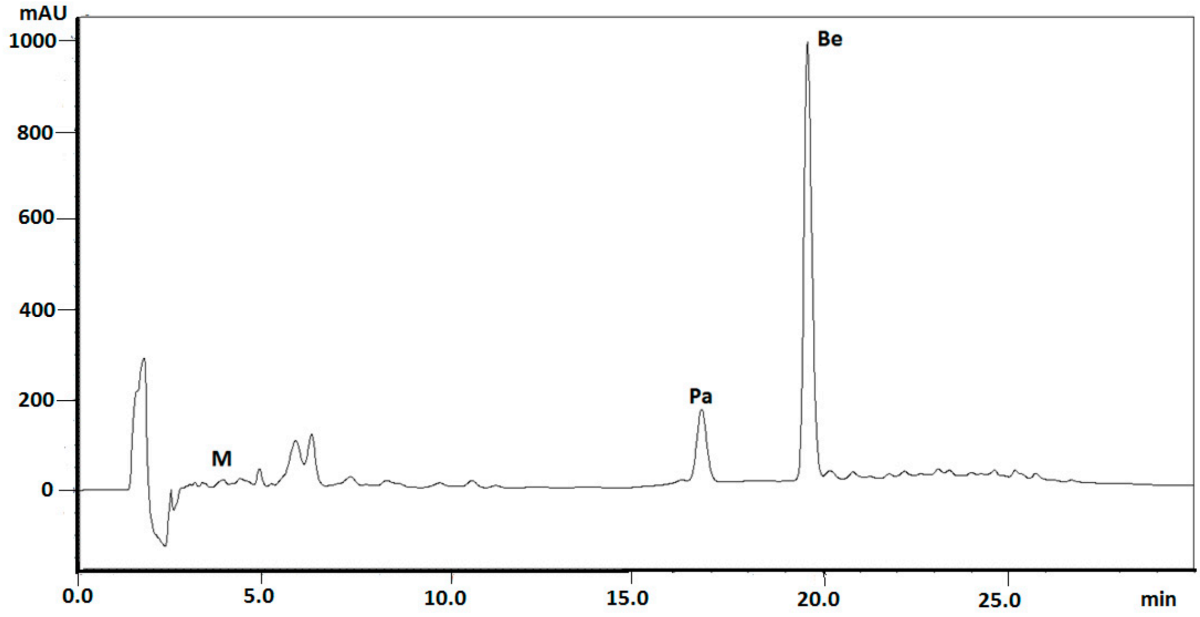

Figure S1. C. Chromatogram obtained for *Mahonia aquifolium* cortex extract from plant material collected during flowering. Abbreviations: M-magnoflorine, Pa-palmatine, Be-berberine.

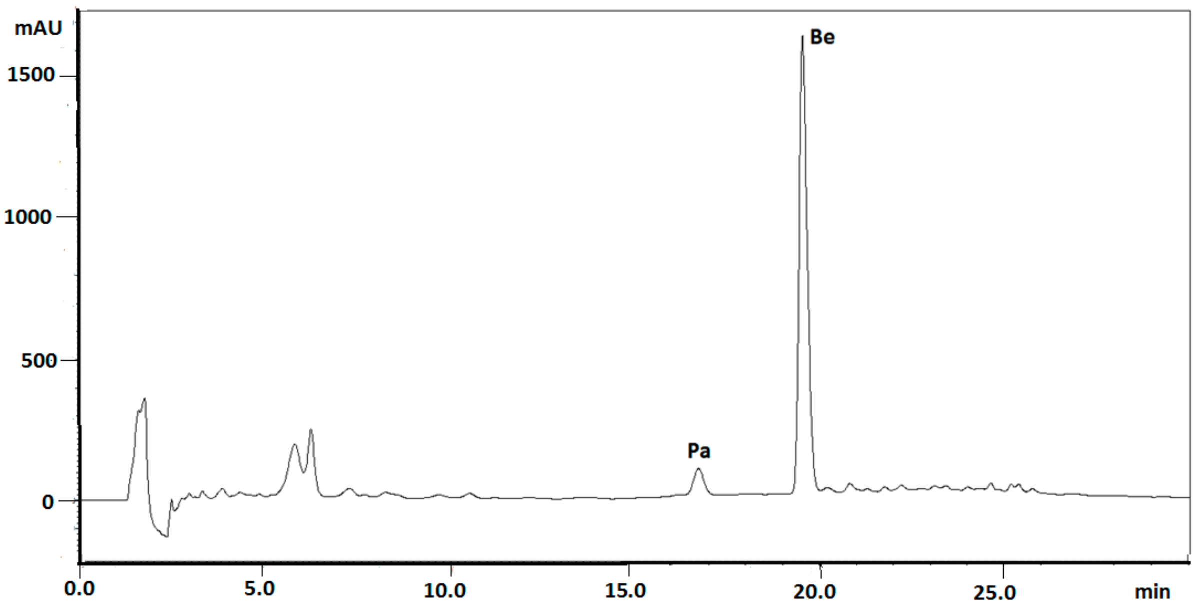

Figure S1. D. Chromatogram obtained for *Mahonia aquifolium* root extract from plant material collected during flowering. Abbreviations: M-magnoflorine, Pa-palmatine, Be-berberine.

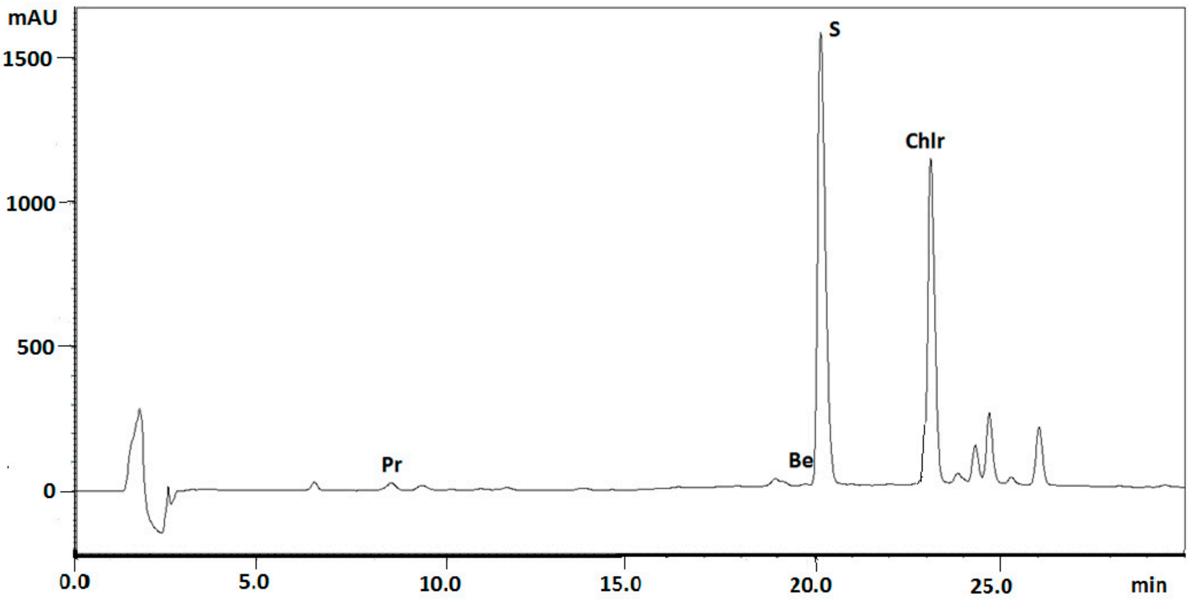

Figure S1. E. Chromatogram obtained for *Sanguinaria canadensis* extract from plant material collected during flowering. Abbreviations: Pr-protopine, Be-berberine, S-sanquinarine, Chlr-chelerythrine.

a)

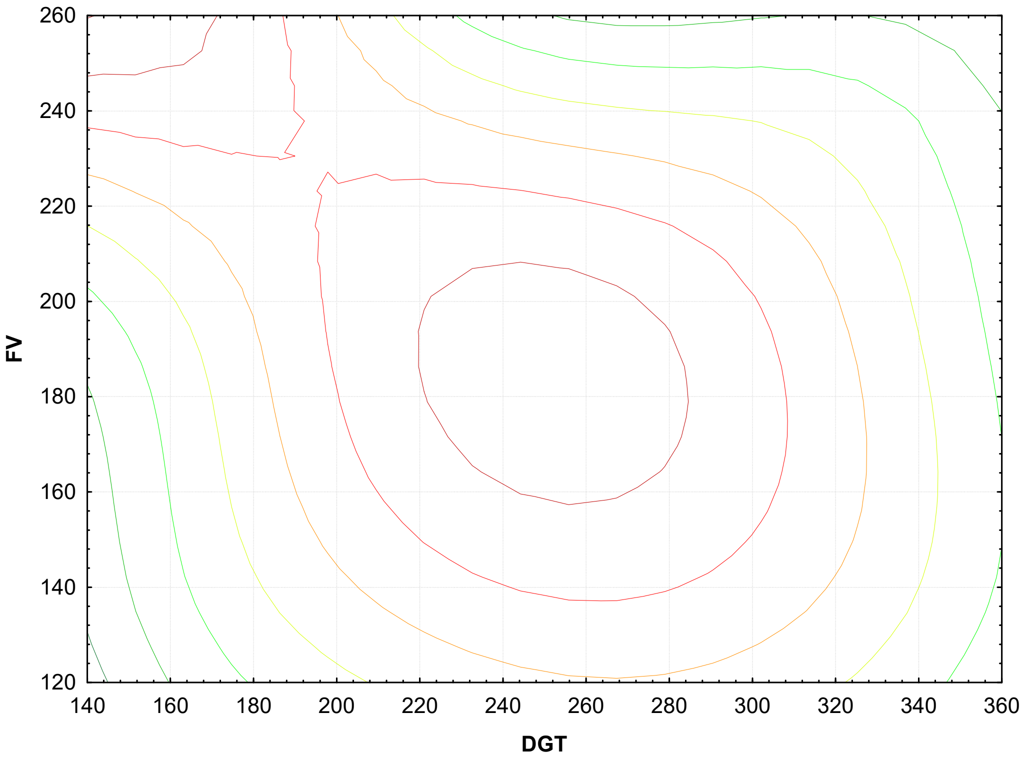

260
240
220
200
180
160
140
120
FV
140 160 180 200 220 240 260 280 300 320 340 360
DGT

(b)

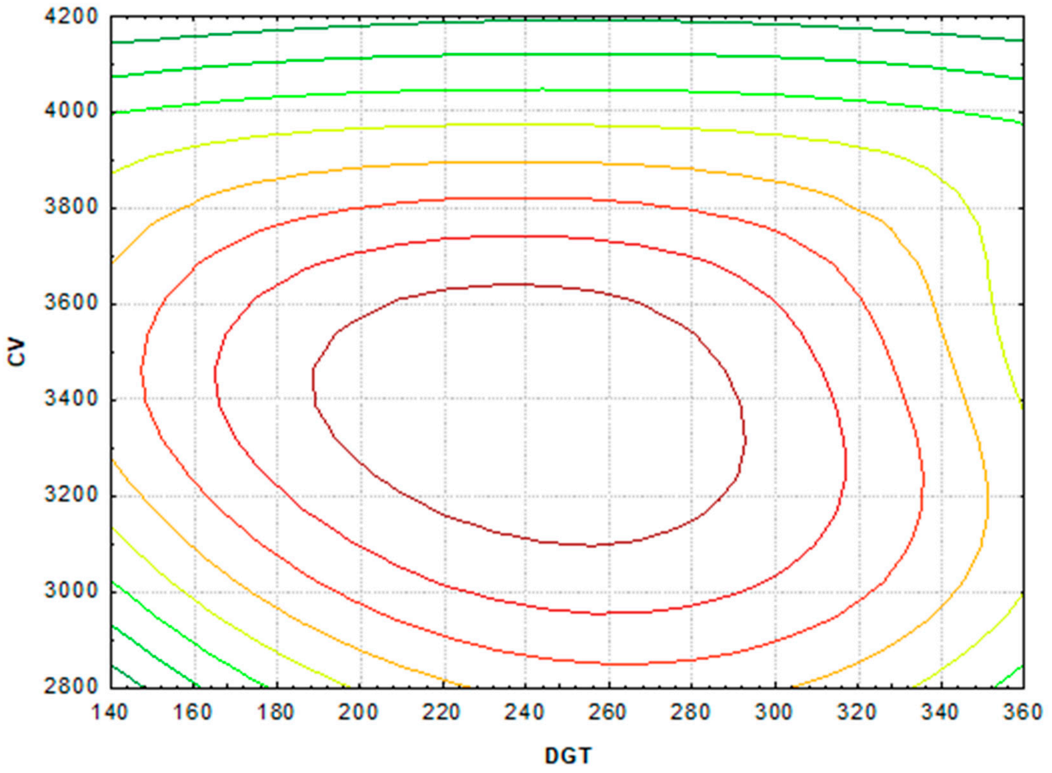

4200
4000
3800
3600
3400
3200
3000
2800
CV
140 160 180 200 220 240 260 280 300 320 340 360
DGT

(c)

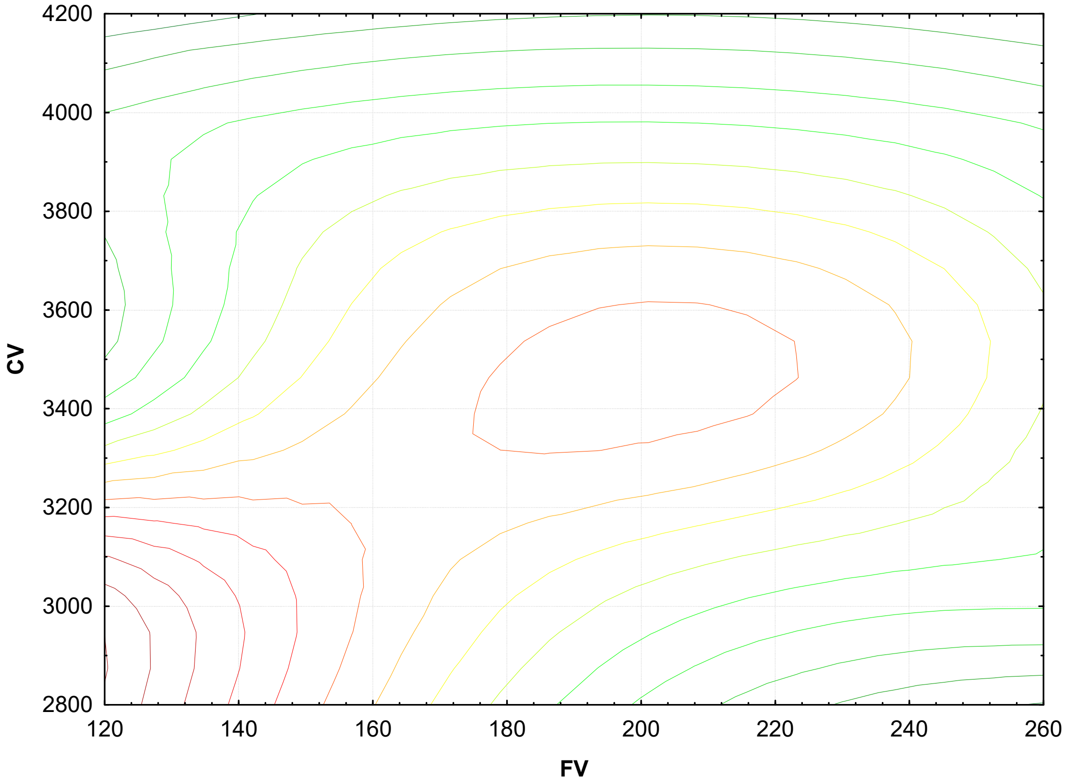

Figure S2. Exemplary of 2D contour plots obtained plotting Box-Behnken for Q-TOF-MS optimization for berberine. Colored zones contain points where the Y function (peak area) fall in the same range, (a) FV *vs.* DGT is plotted; (b) CV *vs.* DGT is plotted; (c) CV *vs.* FV is plotted.

a)

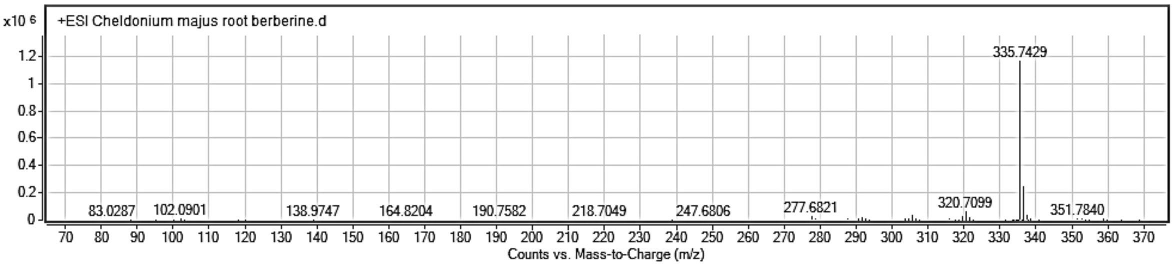

b)

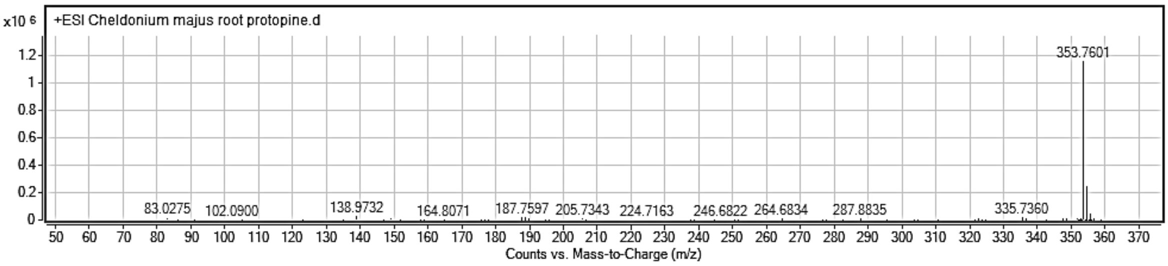

c)

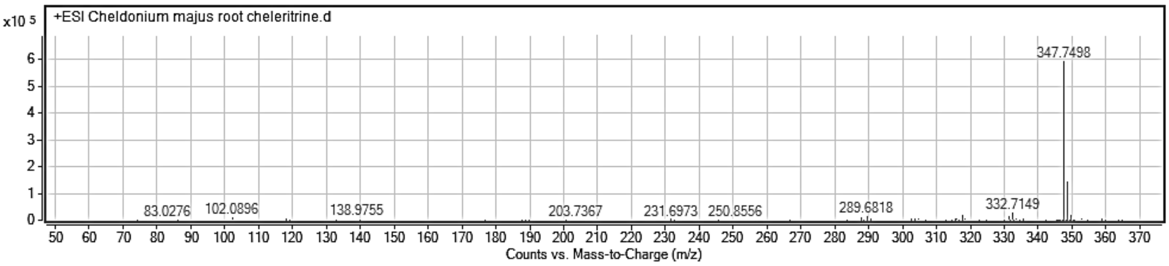

d)

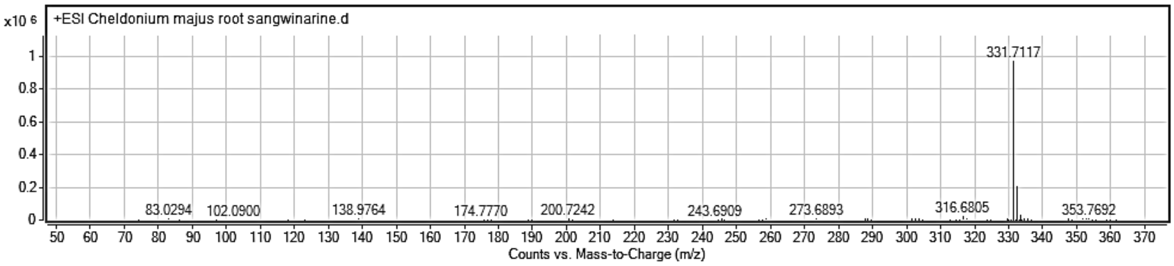

Figure S3. Exemplary of MS spectra obtained for (a) berberine, (b) protopine, (c) chelerythrine,. (d) sanguinarine from *Chelidonium majus.*
